# Polymerases and DNA Repair in Neurons: Implications in Neuronal Survival and Neurodegenerative Diseases

**DOI:** 10.3389/fncel.2022.852002

**Published:** 2022-06-30

**Authors:** Xiaoling Li, Guanghui Cao, Xiaokang Liu, Tie-Shan Tang, Caixia Guo, Hongmei Liu

**Affiliations:** ^1^Nano-Biotechnology Key Lab of Hebei Province, Yanshan University, Qinhuangdao, China; ^2^State Key Laboratory of Membrane Biology, Institute of Zoology, University of Chinese Academy of Sciences, Chinese Academy of Sciences, Beijing, China; ^3^Institute for Stem Cell and Regeneration, Chinese Academy of Sciences, Beijing, China; ^4^Beijing Institute for Stem Cell and Regenerative Medicine, Beijing, China; ^5^Beijing Institute of Genomics, University of Chinese Academy of Sciences, Chinese Academy of Sciences/China National Center for Bioinformation, Beijing, China

**Keywords:** DNA polymerase, RNA polymerase, DNA repair pathway, neurodegenerative diseases, postmitotic cells

## Abstract

Most of the neurodegenerative diseases and aging are associated with reactive oxygen species (ROS) or other intracellular damaging agents that challenge the genome integrity of the neurons. As most of the mature neurons stay in G0/G1 phase, replication-uncoupled DNA repair pathways including BER, NER, SSBR, and NHEJ, are pivotal, efficient, and economic mechanisms to maintain genomic stability without reactivating cell cycle. In these progresses, polymerases are prominent, not only because they are responsible for both sensing and repairing damages, but also for their more diversified roles depending on the cell cycle phase and damage types. In this review, we summarized recent knowledge on the structural and biochemical properties of distinct polymerases, including DNA and RNA polymerases, which are known to be expressed and active in nervous system; the biological relevance of these polymerases and their interactors with neuronal degeneration would be most graphically illustrated by the neurological abnormalities observed in patients with hereditary diseases associated with defects in DNA repair; furthermore, the vicious cycle of the trinucleotide repeat (TNR) and impaired DNA repair pathway is also discussed. Unraveling the mechanisms and contextual basis of the role of the polymerases in DNA damage response and repair will promote our understanding about how long-lived postmitotic cells cope with DNA lesions, and why disrupted DNA repair contributes to disease origin, despite the diversity of mutations in genes. This knowledge may lead to new insight into the development of targeted intervention for neurodegenerative diseases.

## Introduction

The genomes are constantly insulted by reactive molecules, including exogenous source agents and endogenous intermediate products from metabolism, for instance, ultraviolet (UV) light, ionizing radiation (IR), heavy metals, air pollutants, chemotherapeutic drugs, and reactive oxygen species (ROS). These lesions range from modified bases (bulky and non-bulky), abasic sites, inter/intra-strand crosslinks (ICLs), diverse strand breaks, and DNA protein adducts. Among these lesions, oxidized bases account for 1/10 of the total lesions. The nervous system is more vulnerable to oxidative stresses, mainly because it consistently withstands extensive oxidative attacks, which are produced by levels of oxidative metabolism (Narciso et al., 2016). Persistent DNA damage can induce instability in dividing cells and evoke apoptosis signaling in non-dividing cells, thus initiating cancer, aging, and neurodegeneration. In that way, accurate and timely DNA repair processes are essential to counteract DNA damage and restore genomic stability, which in turn guarantee normal cellular activity.

Though DNA repair is such a complex and tightly regulated mechanism in which numerous proteins are involved to address each type of DNA damage in a tissue/cell-specific manner, all of DNA repair pathways require polymerases (Loeb and Monnat, 2008). Mammalian cells express at least 16 DNA polymerases and 3 RNA polymerases that participate in a variety of specialized DNA/RNA synthesis transactions. Polymerases are in such a crucial position that in most cases, the mutations of polymerases are lethal to the organism, or they induce severe developmental syndrome involved in multiple system disorders. Therefore, it is rare that polymerase mutation results in neuronal loss only, and even a tiny disturbance on the pathway that it involved in, would lead to diseases, such as cancer and neurodegeneration. Here, we depicted the role of polymerases in the cell type-specific DNA repair and further discussed why the mutation of the partners, regulators, and the targets of polymerase could act as the source of neurodegeneration and finally elucidated the mutual interaction between gene mutation and the impaired DNA repair pathways.

## Features of Postmitotic Neuron Cells

Nerve cells are unique as they need to survive and preserve their functional complexity for the entire lifetime of the organism, and failure at any level of their supporting mechanisms leads to a wide range of neurodegenerative conditions. Differentiated neurons are postmitotic cells, which are completely devoid of replicative capability. Most mammalian CNS neurons enter the postmitotic state during the embryonic period. Postmitotic neurons are believed to stay in an “extended G0 phase.” They are, however, absolutely incapable of dividing even in the presence of chemical and physical stimuli that promote cell cycle progression in reversible G0 cells. Although this feature is indispensable for the maintenance of once fixed neuronal circuitry, neurons cannot regenerate even under pathological conditions, such as in Alzheimer's disease (AD), which is characterized by marked neuronal degeneration and death (reviewed in Aranda-Anzaldo and Dent, 2017). Neurons display high rates of transcription and translation, which are associated with high rates of metabolism and mitochondrial activity. The amount of oxygen consumed by the brain, that is estimated about 20% of the oxygen consumed by our body far, exceeds that of other organs. This high activity coupled with high oxygen consumption creates a stressful environment for neurons: damaging metabolic byproducts, primarily reactive oxygen species (ROS), are constantly attacking neuronal genomic and mitochondrial DNA reviewed in Lennicke and Cochemé, 2021). It is reported that ROS is about 1–2% of the total oxygen consumption per day (Harman, 1956). Hence, the genomic DNA in brain cells is shown to suffer various types of damage, including oxidative damage and double-strand breaks, even in the absence of any specific environmental insult (Suberbielle et al., 2013; Talhaoui et al., 2017; Hou et al., 2019).

Since neurons are irreplaceable and should survive as long as the organism does, they need elaborate, stringent defense mechanisms to maintain their high metabolic activity and gene expression for the sake of their longevity.

## Source of Endogeneous and Exogenous DNA Damage to Postmitotic Neurons

DNA constantly faces attacks from both exogenous and endogenous sources.

Radiation, chemotherapeutic reagents, and environmental pollutants are considered as common exogenous sources of DNA damage. Radiation, such as X, γ, and cosmic rays, radon decay, leads to various types of direct phosphodiester strand breaks DNA-strand breaks. The chemotherapeutic agents and environment pollutants form chemical-base adducts and further generate “simple” and helix-distorting base lesions, crosslinks, and strand breaks. The central nervous system, which is protected by the skin, skull, spine and the blood–brain/spinal cord barrier, is free from UV, most environment pollutants and chemotherapeutic agents that are used for the therapy of cancers, but remains vulnerable to radiation and certain chemicals, for example, temozolomide and irinotecan (Juillerat-Jeanneret, 2008).

The endogenous sources, generally including spontaneous hydrolysis of DNA, intracellular metabolites, and oxidative stresses, lead to the formation of abasic (or apurinic/apyrimidinic, AP) sites or inappropriate base entities, alkylated adducts, and numerous DNA backbone and base oxidative modifications. Under normal conditions, the estimated number of DNA lesions caused by endogenous sources was recently estimated at ≥50,000 lesions per cell; the non-instructional and pro-mutagenic APs are the most common DNA lesions, present daily at ~30,000 nucleoside sites in DNA per cell (Klapacz et al., 2016).

Notably, reactive oxygen species (ROS) contributes to most of the endogenous source of DNA damage that occurs in the brain, due to the brain's high demand for energy (Lennicke and Cochemé, 2021). The intracellular generation of reactive oxygen species (ROS), such as superoxide anion radicals (O2•-), hydrogen peroxide (H_2_O_2_), and hydroxyl radicals (•OH), represents an additional, significant source of endogenous DNA oxidation by several different mechanisms and induces different types of lesions, including AP, oxidized bases [such as 7,8-dihydro-8-oxoguanine (8-oxoG) and 8-hydroxy-2-deoxyguanosine (2-doxG)], cyclopurines, and SSBs (reviewed in Talhaoui et al., 2017). There are more than 80 different types of base and sugar lesions induced by ROS identified. Among these, the major endogenous oxidized bases are 8-oxo-7,8-dihydroguanine (8-oxoG), which is mutagenic. In fact, oxidized purine at C5 atom, the 5-hydroxyuracil (5ohU) and 5-hydroxycytosine (5ohC), are also miscoding (Grollman and Moriya, 1993). Oxidation of adenine generates 2-hydroxyadenine (2-oxoA), 8-oxo-7,8-dihydroadenine (8-oxoA) and formamidopyrimidine (Fapy). It should be noted that adenine modifications, including 8-oxoA and FapyA, are about 10-folds lower than that of guanine upon exposure to ROS (Pang et al., 2014). The increased level of these small modifications is reported in nearly all neurodegenerative diseases (reviewed in Coppedè and Migliore, 2015; Zuo et al., 2021).

In addition to small base modifications that are instanced above, ROS can also generate bulky adducts, such as (5'S)- and (5'R)-8,5'-cyclo-2'-deoxyadenosine (cdA), 8,5'-cyclo-2'-deoxyguanosine (cdG), 5',8-cyclo-2'-deoxyribonucleosides (cdPu), thymine glycol, and G[8-5 m]T intra-strand crosslinks. cdA induces large changes in backbone torsion angles of DNA duplex, which strongly perturbs the helix conformation near the lesion, and therefore becomes strong blocks for both DNA replication and transcription (Kuraoka et al., 2000; Weng et al., 2018; Tsegay et al., 2022). Though cdA and cdPu could be barely detectable in wildtype mouse brain (Wang et al., 2012), they accumulate to a high level in the kidney, liver, and brain of CSB knockout (KO) animals and in the brain of XPA KO mice in an age-dependent manner (Kirkali et al., 2009; Mori et al., 2019), highlighting the importance of ROS in the pathogenesis of neurological abnormalities. One possible reason that why general neuron vulnerability to ROS damages is their high metabolic activity and reliance on oxidative phosphorylation over glycolysis as their main source of energy, which leads to increased generation of reactive oxygen species and consequently leads to increased oxidative DNA damage. A factor that could compound this effect is the mitotic status of neurons as it has previously been suggested that postmitotic cells are more likely to accumulate DNA damage than mitotic cells. For example, it has been shown that postmitotic parenchymal liver cells exhibit an age-related increase in alkali-labile sites that is not observed in mitotically active non-parenchymal liver cells (Mullaart et al., 1988). Yet, we still cannot tell whether the increased level of ROS is a causative agent or the consequence of numerous neurodegenerative diseases.

Besides oxidation, endogenous DNA alkylation adducts contribute another main part of the total background levels of all DNA adducts present at steady-state levels in cells (Soll et al., 2017; Sobol, 2021). Endogenous alkylating DNA adducts can arise from several different sources, for example, from metabolic activity of gut bacteria, or as byproducts of lipid peroxidation, or reacting with cellular methyl donors, such as S-adenosylmethionine, a common cofactor in cellular methylation reactions (Taverna and Sedgwick, 1996). The most and second abundant adduct produced by these alkylating agents is N7-methylguanine (N7-MeG) and N3-methyladenine (N3-MeA) adducts, which are non-cytotoxic and highly cytotoxic but slightly mutagenic, respectively (Yoon et al., 2017; Koag et al., 2019). In double-strand DNA, O6-methylguanine (O^6^-MeG) are also prevalent, that are the major pro-mutagenic adducts to induce G:C-to-A:T mutations and highly cytotoxic (Wang et al., 2019). N7-MeG and O^6^-MeG are reported to closely relate to the onset of Western Pacific Amyotrophic lateral Sclerosis (WP-ALS). There is a correlation of the incidence between WP-ALS and the use of traditional foods or medicines containing material from local cycad seeds, which contain neurotoxins, including methylazoxymethanol (MAM), β-N-methylamino-L-alanine (BMAA), and β-sitosterol β-d-glucoside (Vega and Bell, 1967; Khabazian et al., 2002), which could induce N7-MeG and O6-MeG in rat cortical neurons, and motor impairment and/or motor neuron abnormalities in mice (Esclaire et al., 1999; Kisby et al., 2011), and associated increases in γH2AX expression and genomic instability (Chiu et al., 2012; Gerić et al., 2019), suggesting a correlation between alkylation of DNA and motor neuron degeneration and ALS-like symptoms through DNA damage (Kok et al., 2021).

It should be noticed that 5-methylcytosine (5mC) and 5-hydroxymethylcytosine (5hmC), the hydroxylated form of 5mC, in CpG islands, are important epigenetic retouching for gene expression regulation and imprinting in brain and embryonic stem cells (Li and Zhang, 2014). 5hmC is enriched in the nervous system and displays neurodevelopment and age-related changes in particular (Bernstein et al., 2016). The abnormal level of 5hmC is reported to increase significantly in the middle frontal gyrus and middle temporal gyrus of patients with AD (Coppieters and Dragunow, 2011), implicating the importance of the regulation of cytosine methylation. Notably, the demethylation of cytosine 5mC is also more active in postmitotic neurons than in peripheral cell types, e.g., the hyperactivity of demethylation in GABAergic and glutamatergic neurons is associated with of schizophrenia (SZ) and bipolar (BP) disorder. Demethylation *via* members of the TET family of proteins in mammalian brains is initiated through progressive oxidation of 5mC to 5hmC, 5-formylcytosine (5fC), or 5-carbolxylcytosine (5caC), and steady-state levels of 5hmC account for approximately 40% of modified cytosines in the brain (Wu and Zhang, 2014). Then, 5fC and 5caC are specifically recognized by thymine deglycosylase (TDG) producing APs. It is possible that cycles of cytosine methylation and demethylation are the potential sources of neuronal site-specific DNA single-strand breakage (Wu et al., 2021).

Though the methylation of cytosine is typical endogenous methylation products, the levels of 5mC and 5hmC are affected by environment, disease, age, and gender and are undergoing dynamic changes as the results of both *de novo* DNA methylation and demethylation (Dong et al., 2017; Jin and Liu, 2018; Ao et al., 2022). As a matter of fact, since major exogenous sources of alkylating agents come from natural and anthropogenic constituents of air, water, and food, as well as from tobacco smoke and fuel combustion products, it is difficult to distinguish a small risk at low-dose exposures within the normal distribution of the background range of mutation. For instance, catechol quinones are the oxidation metabolites of natural estrogens and dopamine, yet reported to form stable estrogen adducts with N6 dA, N7 dG and N3 nucleobase Ade (Cavalieri et al., 2002, 2004). The rapidly depurinating N3Ade adducts then result in a burst of apurinic sites that overwhelm the repair machinery of the cell, and this DNA damage may be at the origin of Parkinson's and other neurodegenerative diseases.

Moreover, the complexity of DNA damage is that one form of lesion can be turned into another form. For example, AP, if not repaired, can be converted to SSBs. Oxidative and alkylation of DNA always coupled with the generation of SSBs, and the persistent unrepaired bi-stranded oxidative and alkylation damages in close proximity could result in secondary double-strand breaks (DSBs) (Abbotts and Wilson, 2017; Soll et al., 2017). DSB accumulation plays a crucial function in cell cycle re-entry in the postmitotic neuronal cells (van Leeuwen and Hoozemans, 2015). For instance, a study reported that in AD brain samples, the accumulation of DSB leads to the formation of inactive monomers and dimers of TP53, which causes neuronal cell cycle re-entry and subsequent cell death (Katsel et al., 2013).

Here, we listed the DNA adducts and lesions attacked by exogenous and endogenous sources that the neurons commonly face in Table 1.

**Table 1:** Examples of DNA lesions that stalls RNA Pol II.

**DNA lesions**	**DNA repair pathways**	**Effect on transcription**	**TFIIS**	**DSIF (Spt4/5)**	**CSB (Rad26)**	**Response of RNA polymerase II**	**Detectable in brain**
**Small DNA lesions** 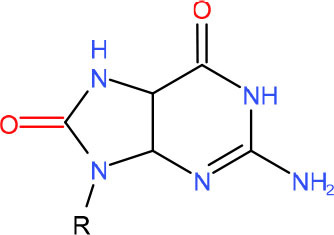 **8-Oxoguanine (8-oxo-G)**	• BER in mitotic/postmitotic cells, NER as the backup of BER (Boiteux et al., 2017) • Translesion synthesis in postmitotic cells (Shibutani et al., 1991; Charlet-Berguerand et al., 2006)	Weak pausing (Charlet-Berguerand et al., 2006; Oh et al., 2021)	• **(++)** (Kim et al., 2006; Brégeon and Doetsch, 2011) • (–) (Liu et al., 1995; Charlet-Berguerand et al., 2006; Kornberg, 2007)	N.D.	**(++)** (Charlet-Berguerand et al., 2006; Kim et al., 2006; Brégeon and Doetsch, 2011)	Quick bypass and transcriptional mutagenesis (~50–80%) (Charlet-Berguerand et al., 2006; Oh et al., 2021)	Y
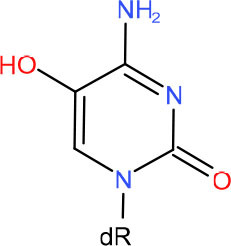 **5-Hydroxycytosine (5-OH-C)**	BER in mitotic/postmitotic cells (Charlet-Berguerand et al., 2006; Gasparutto et al., 2009)	Weak pausing (Charlet-Berguerand et al., 2006)	(–)	N.D.	(–)	Quick bypass and transcriptional mutagenesis (~40–60%) (Charlet-Berguerand et al., 2006)	Y (Charlet-Berguerand et al., 2006; Lovell and Markesbery, 2007)
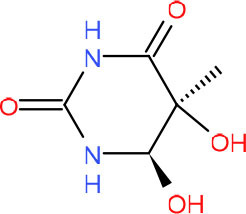 **Thymine glycol (TG)**	• BER in mitotic/postmitotic cells (Kuznetsova et al., 2020; Chakraborty et al., 2021) • NER	• Strong pausing • Weak pausing (Tornaletti et al., 2001)	(–)	N.D.	**(+)** (Spivak and Hanawalt, 2006)	Slow bypass and transcriptional mutagenesis (~20–60%)	Y (Canugovi et al., 2013)
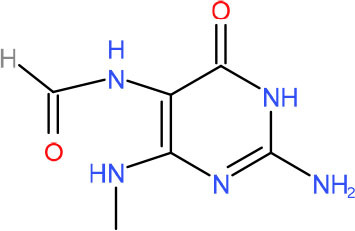 **Formamidopyrimidine lesions (FapyG)**	BER in mitotic/postmitotic cells (Canugovi et al., 2013)	N.D.	N.D.	N.D.	N.D.	N.D.	Y (Canugovi et al., 2013)
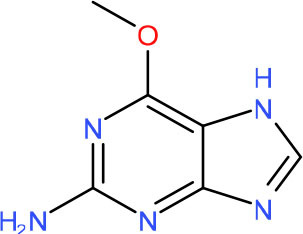 **O6-methyl guanine (O6-Me-G), derived from cycad seed and endogenous alkylating agents**	• MGMT/MMR and TLS in mitotic cells(Nay and O‘Connor, 2013) • MGMT/BER in postmitotic cells (Kaina et al., 2001)	• Weak pausing (anti-proximal); • Strong pausing (other conformation) (Dimitri et al., 2008)	N.D.	N.D.	(–) (Vitaly Latypov et al., 2012)	• Quick bypass (anti-proximal) and transcriptional mutagenesis; • Initiate TC-NER (other conformation) (Dimitri et al., 2008; Vitaly Latypov et al., 2012)	Y (Kisby et al., 2011)
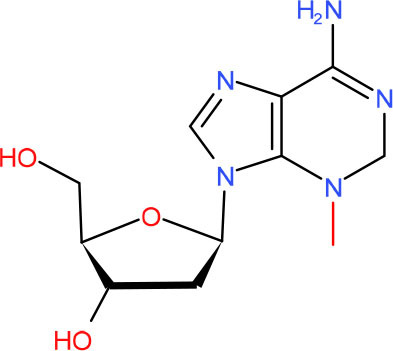 **N3-methyl-adenine (3mA), main adduct from MMS and endogenous alkylating agents**	• BER (O'Brien and Ellenberger, 2004a,b; Grøsvik et al., 2020) • NER (Scicchitano and Hanawalt, 1989; Plosky et al., 2002)	Weak pausing (Malvezzi et al., 2017)	N.D.	N.D.	N.D.	Quick Bypass and transcriptional mutagenesis (Malvezzi et al., 2017)	Y (Ohba et al., 2021)
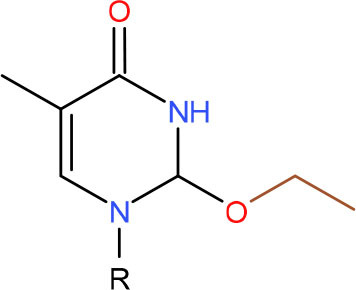 **O2-ethylthymidine (O2-ET), smoke-related adduct**	Unrepaired (Den Engelse et al., 1987; Bronstein et al., 1992)	Strong pausing (You et al., 2014)	(– –) (Xu L. et al., 2017)	N.D.	N.D.	Transcriptional mutagenesis slow bypass and initiation of TC-NER (You et al., 2014)	Y (Mientjes et al., 1996; Wang et al., 2016)
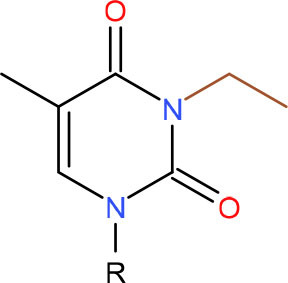 **N3-ethylthymidine (N3-ET), smoke-related adduct**	BER in postmitotic cells (You et al., 2016)	Strong pausing (You et al., 2014)	(– –) (Xu L. et al., 2017)	N.D.	N.D.	Transcriptional mutagenesis slow bypass and initiation of TC-NER (You et al., 2014)	Y (Mientjes et al., 1996; Wang et al., 2016)
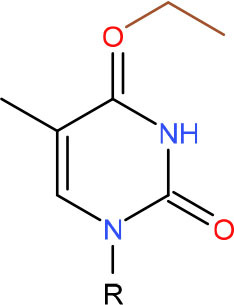 **O4-ethylthymidine (O4-ET), smoke-related adduct**	Unrepaired (Den Engelse et al., 1987; Bronstein et al., 1992)	Weak pausing (You et al., 2014)	(–) (Xu L. et al., 2017)	N.D.	N.D.	Quick bypass and transcriptional mutagenesis (You et al., 2014)	Y (Mientjes et al., 1996; Wang et al., 2016)
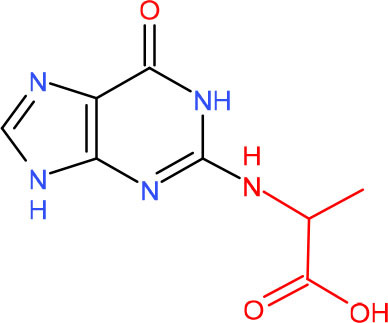 **N2-(1-Carboxyethyl)-guanine (CEG)**	TLS in mitotic cells (Yuan et al., 2011)	Strong pausing (You et al., 2012; You and Wang, 2016)	N.D.	N.D.	N.D.	TC-NER initiation (You et al., 2012; You and Wang, 2016)	N.D.
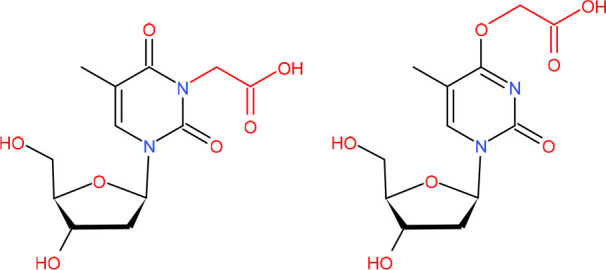 **Left: N3-carboxymethyl-thymidine N3-(CMT)** **Right: O4-carboxymethylthymidine (O4-CMT)**	TLS in mitotic cells (Wu et al., 2017)	Strong pausing (You et al., 2015)	N.D.	N.D.	N.D.	Slow bypass and transcriptional mutagenesis (You et al., 2015)	N.D.
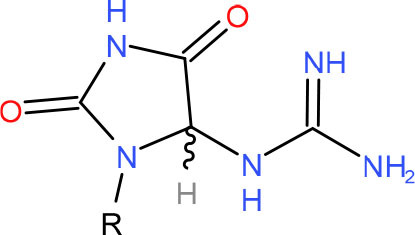 **5-Guanidinohydantoin (Gh)**	• BER (Krokeide et al., 2013; Shafirovich et al., 2016) • NER (Shafirovich et al., 2016)	Strong pausing (Kolbanovskiy et al., 2017; Oh et al., 2020)	(–) (Oh et al., 2020)	N.D.	(–) (Oh et al., 2020)	• Initiation of TC-NER (Kolbanovskiy et al., 2017) • Slow error-prone incorporation of purines (Oh et al., 2020)	Y (Christine Regnell et al., 2012)
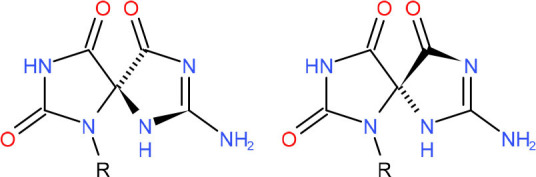 **R-(left) and S-(right) Spiroiminodihydantoin (Sp)**	• BER (Krokeide et al., 2013; Shafirovich et al., 2016) • NER (Shafirovich et al., 2016)	Strong pausing (Kolbanovskiy et al., 2017; Oh et al., 2020)	(–) (Oh et al., 2020)	N.D.	(–) (Oh et al., 2020)	• Initiation of TC-NER (Kolbanovskiy et al., 2017) • Slow error-prone incorporation of purines (Oh et al., 2020)	Y (Christine Regnell et al., 2012)
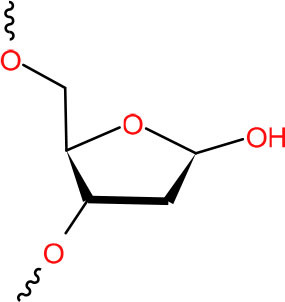 **Abasic site**	BER (Quiñones et al., 2020)	Strong pausing (Wang et al., 2018a)	(– –) (Owiti et al., 2017)	N.D.	N.D.	Slow bypass and transcriptional mutagenesis (Wang et al., 2018a)	Y (Lovell and Markesbery, 2007)
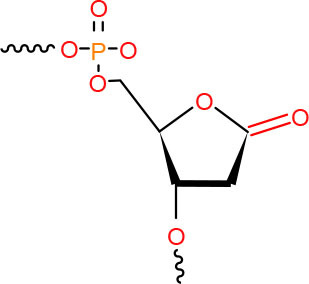 **2-Deoxyribonolactone (dL), oxidized form of AP site**	BER (Sung and Demple, 2006)	Strong pausing (Wang et al., 2006)	(– –) (Wang et al., 2006)	N.D.	N.D.	TC-NER initiation (Wang et al., 2006)	N.D.
**Bulky and helix-distorting DNA lesions** 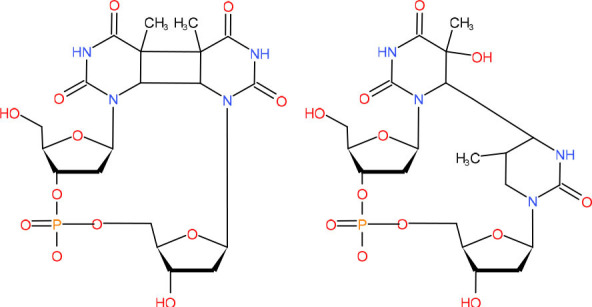 **Left: Cyclobutane pyrimidine dimer (CPD)** **Right: 6–4 UV photoproduct ((6-4)-PP)**	GG-NER in mitotic cells and poor repaired by TC-NER in postmitotic cells (van der Wees et al., 2003)	Strong pausing (Mei Kwei et al., 2004; Oh et al., 2020)	(– –) (Labhart, 1997; Kalogeraki et al., 2005; Charlet-Berguerand et al., 2006; Xu J. et al., 2017; Xu L. et al., 2017)	N.D.	• (–) (Labhart, 1997; Xu J. et al., 2017) • (+)	• Slow bypass and transcriptional mutagenesis (Marietta and Brooks, 2007; Walmacq et al., 2012) • TC-NER initiation (Brueckner et al., 2007)	N
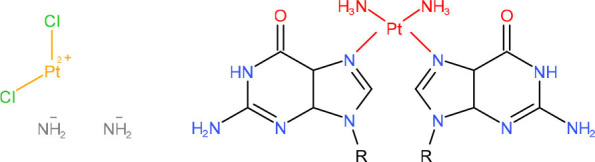 **Left: Cisplatin** **Right: 1,2-dGpG Cisplatin DNA Adduct**	NER in mitotic cells and TC-NER in postmitotic cells (Wang and Zhu, 2018)	Strong pausing (Jung and Lippard, 2003; Tornaletti et al., 2003; Damsma et al., 2007)	• (–) (Damsma et al., 2007) • (– –) (Tornaletti et al., 2003; Tremeau-Bravard et al., 2004)	N.D.	(–) (Tremeau-Bravard et al., 2004)	TC-NER initiation (Damsma et al., 2007)	N (Juillerat-Jeanneret, 2008)
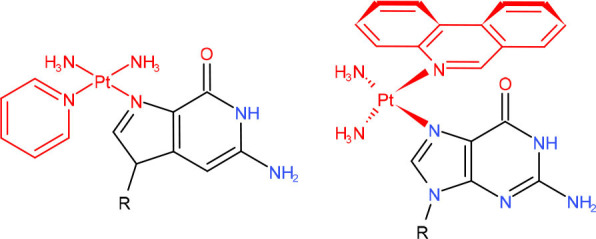 **Left: Pyriplatin-dG** **Right: Phenanthriplatin-dG**	TC-NER (Wang et al., 2018b)	Strong pausing (Wang et al., 2010, 2015; Kellinger et al., 2013)	N.D.	N.D.	N.D.	Lesion bypass with/without transcriptional mutagenesis (Kellinger et al., 2013; Wang et al., 2015)	N.D.
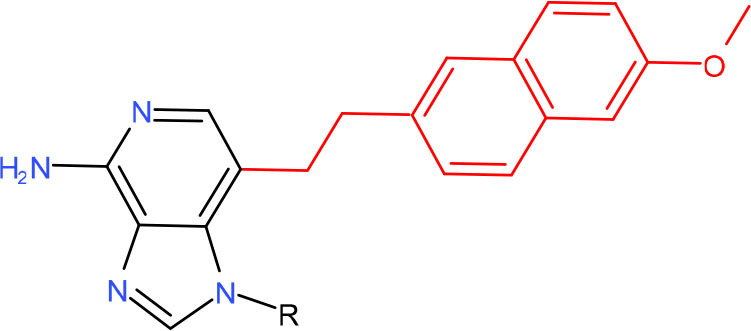 **3-deaza-3-methoxynaphtylethyl-adenosine (3d-Napht-dA), main adduct from HMAF**	TC-NER (Wang et al., 2018b)	Strong pausing (Malvezzi et al., 2017)	N.D.	N.D.	N.D.	N.D.	N.D.
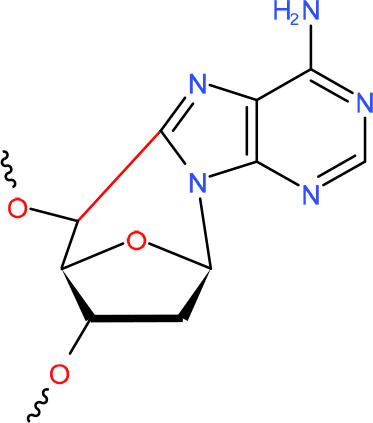 **CydA**	NER (Brooks, 2017)	Strong pausing (You et al., 2012)	N.D.	N.D.	N.D.	Slow bypass and transcriptional mutagenesis (Marietta and Brooks, 2007)	Y (Wang et al., 2012)
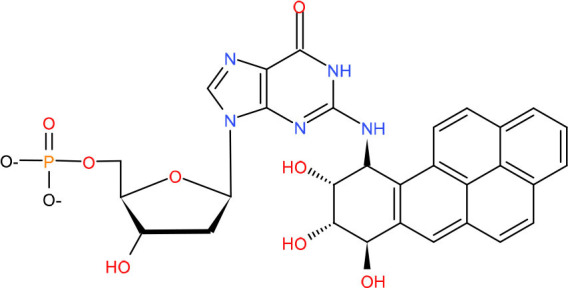 **benzo[a]pyrene diol epoxide (BPDE)**	NER and BER(Braithwaite et al., 1998)	Strong pausing (Remington et al., 1998; Perlow et al., 2002)	N.D.	N.D.	• (–) (Wijnhoven et al., 2000; Lagerqvist et al., 2008) • (– –) (Leng et al., 2008)	• Lesion bypass and transcriptional mutagenesis for 32% of the (–)-anti-trans-BPDE DNA adduct and 18% for the (+)-anti-trans-BPDE (Remington et al., 1998; Perlow et al., 2002); • Initiation of TC-NER (Lagerqvist et al., 2008; Leng et al., 2008)	Y (Chepelev et al., 2015)
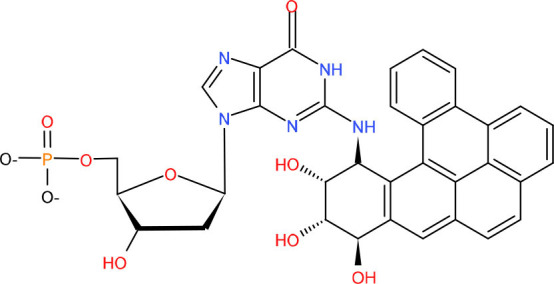 **dibenzo[a,l]pyrene diol epoxide (DBPDE)**	Replication bypass (Lagerqvist et al., 2008)	Strong pausing (Zhong et al., 2010)	N.D.	N.D.	(– –) (Zhong et al., 2010)	Initiation of TC-NER (Zhong et al., 2010)	N.D.

(–) *Little or no effect;*

(– –) *Prevent bypass;*

(+) *Promote bypass (moderate effect);*

(++) *Promote bypass (strong effect); N.D., not determined*.

## DNA Repair Pathways that are Activated in Postmitotic Neurons

To postmitotic neurons, who are in absence of replication but active in transcription and translation, removal of DNA from the nonessential bulk of their genome is dispensable, and these cells can afford to repair only the portion of the genome needed for their functions, e.g., their transcribed DNA (Banerjee et al., 2011). Therefore, DNA repair systems, such as nucleotide excision repair (NER), base excision repair (BER), and single-strand break repair (SSBR), are largely enhanced (Li et al., 2019; Chakraborty et al., 2021); conversely, replication-derived repair, such as mismatch repair (MMR), translesion synthesis (TLS) is not possible in these cells.

It is assumed that most of the small lesions inflicted in neuronal genomic and mitochondrial DNA by ROS, the principal damaging agent in these cells, are typically repaired *via* the BER pathway, and this activity has been documented in the nuclear and mitochondrial compartments (D'Errico et al., 2021). As AP sites are naturally produced in special DNA regions in neurons (Wu et al., 2021), BER is of significant importance in maintaining the genome integrity and phycological functions of neurons. Importantly, SSBs are also intermediate products of BER, and thus, there is significant overlap between SSB repair (SSBR) and BER pathways (Antoniali et al., 2014). An increasing number of evidence in defective BER pathway machinery have been linked to various neurological diseases; for instance, spinocerebellar ataxia with axonal neuropathy-1 (SCAN1) and ataxia-oculomotor apraxia-1 (AOA1) are caused by mutations in two peripheral SSBR proteins AOA1 and tyrosyl-DNA phosphodiesterase 1 (TDP-1), clearly showing the importance of BER in human neuronal maintenance (El-Khamisy et al., 2005; Ahel et al., 2006). In addition, studies of BER capacity in brain tissue from patients with sporadic AD and mild cognitive impairment (MCI) and normal age-matched controls indicated an impairment of BER activity, which was inversely correlated with the severity of these diseases (Lopez-Gonzalez et al., 2016; Ravel-Godreuil et al., 2021).

Compared to BER, which is predominant for the processing small base modifications, such as alkylation, deamination, and base oxidation, nucleotide excision repair (NER) pathways remove many diverse helix-distorting lesions and crosslinks caused by UV radiation, chemical agents, and some types of oxidative lesions, cyclopurines, thymine glycol, as well as malondialdehyde and ethylene adducts, for instance (Izumi and Mellon, 2021). Notably, these DNA lesions are not only the substrates for NER but have also been found to block transcription (Duan et al., 2021). NER consists of two subpathways: global genome (GG-NER) and transcription-coupled NER (TC-NER). GG-NER repairs general helix-distorting lesions anywhere in the genome, whereas TC-NER deals with the damages that block transcription and is initiated by stalling of RNA polymerase II (Duan et al., 2021). Since the aim of DNA repair in neurons was to remove all obstacles to transcription and translation, TC-NER is of significant importance to neurons. Correspondingly, evidence suggested that upon terminal differentiation, cells augmented the ability in repairing lesions in transcribed genes, whereas cells lost the ability to repair damages in non-transcriptional regions (Li et al., 2019; Chakraborty et al., 2021). All these results indicated that the TC-NER is probably the predominant NER pathway in neurons. The importance of the pathway is seen from mutations in NER genes leading to rare human diseases, such as Cockayne syndrome (CS) and xeroderma pigmentosum (XP). These diseases have premature aging features, including extensive neurological symptoms (Menck and Munford, 2014). Despite these diseases having progeria symptoms, there is not a consensus whether NER capacity decreases in normal human aging.

Additionally, single-strand break repair (SSBR) and double-strand break repair (DSBR) pathways mend DNA-strand breaks caused by ionizing irradiation, oxidation, and chemotherapy reagents. SSBR is generally considered a specialized, subpathway of BER, since it often engages proteins dedicated to BER (Caldecott, 2014). Double-strand breaks (DSBs) are repaired through one of two mechanisms: nonhomologous end joining (NHEJ) or homologous recombination (HR) repair. HR is the major pathway used during S/G2 phase, where the broken DNA is repaired using the sister chromatid as a template, that is to say HR only occurs in mitotic cells. Counterpart of HR, the NHEJ repair can happen during any phase of cell cycle, and it is the primary means for repairing DSBs in postmitotic neurons. Because the damaged DNA terminals need to be processed before rejoining, errors can be introduced during NHEJ repair.

The role of DSB repair, NHEJ in particular, in the aging process has also been extensively studied, and disruption of genes involved in this pathway leads to progressive and permanent neuronal damage and impairing cognitive and motor functions (Madabhushi et al., 2014; Alt and Schwer, 2018; Khan et al., 2018). Emerging findings suggest that an imbalance between DSBs accumulation and repair in brain contributes to neuronal damage, impaired learning, and memory and has been documented in the pathogenesis of a broad spectrum of human neurodegenerative diseases, including AD, PD, ALS (Wang et al., 2013; Merlo et al., 2016; Milanese et al., 2018; Yu et al., 2018; Wang and Hegde, 2019), and accelerated aging phenotypes, such as those observed in Werner syndrome (WS), Ataxia–telangiectasia (AT) (Oh and Myung, 2022), and immunodeficiency 26 with or without neurologic abnormalities (IMD26) (Woodbine et al., 2013). These findings strongly endorse the possibility that declined NHEJ and unrepaired DSBs significantly contribute to neurodegenerative disorders, and targeting DSB signaling could lead to novel therapeutic routes for attenuating these diseases.

Despite a clear association between DNA damage and neurodegeneration, the DDR and DNA repair have not been extensively compared between different neuron populations, and it remains unclear whether specific neuron types, such as motor neurons, are more vulnerable to DNA damage or DNA repair deficiencies. Notably, the majority of neurodegenerative diseases caused by DDR or DNA repair gene mutations affect cerebellar neurons, especially rather than motor neurons. Moreover, the brain also experienced ongoing DNA damage and repair in a cell type-specific manner under physiological conditions during aging. Specifically, the relative amount of spontaneous nuclear DNA repair in the mouse brain decreased during aging in hippocampal pyramidal and granule cells, as well as in cortical layer V pyramidal cells and neurons in the striatum and thalamus, but not in Purkinje cells, mitral cells in the olfactory bulb, and large neurons in the lateral vestibular nucleus, whereas SSBs accumulated in hippocampal pyramidal and granule cells, as well as cortical layer V pyramidal cells, and neurons in the striatum and thalamus showed an age-related increase in the relative amount of DNA SSB, whereas Purkinje cells did not (reviewed in Brasnjevic et al., 2008). These findings implicated that BER/SSBR defect is responsible for the selective neuronal vulnerability in neurodegenerative diseases. In line with that, mutation/depletion of BER/SSBR components, such as UNG1, OGG1, and XRCC1, resulted in the degeneration of CA3 pyramidal neurons, striatal dopamine neurons, hippocampal pyramidal, and cerebellar granule cells in mice models. A clear picture emerging from the analysis of these cell type specificity is that the cerebellum appears to be the brain region with the highest vulnerability to defects in BER/SSBR activities, corresponding to the high abundance/activity of BER core proteins (OGG1, UNG, and NTHs) and end-processing DNA repair factors, such as APTX and TDP1 in the cerebellum, which could be suggestive of a high susceptibility of this brain region to DNA lesions and especially to oxidative DNA damages. Particularly, cerebellar granule neurons and CA1 neurons are vulnerable to oxidative stress stimuli compared to other neurons, such as cortical and CA3 neurons. It should be noticed that the specific brain regions are associated with the clinical phenotypes of neurodegenerative diseases, reflecting the relationship between BER/SSBR and disease causality (reviewed in Narciso et al., 2016).

It seems that BER/SSBR is responsible for the repair of cognitive-related neurons, whereas NER, TC-NER for special, has been proposed to be more relevant to the restore of DNA damages in motor neurons. A higher degree of neuronal cell death is observed in NER neurodegenerative diseases—the cerebellum of patients with CS, displays the loss of Purkinje cells, and mice with reduced expression of ERCC1, a protein involved in NER, show age-dependent motor neuron degeneration and astrogliosis, similar to amyotrophic lateral sclerosis (ALS) (Goetz, 2000; Kajitani et al., 2021). However, further investigation is required to determine whether the differences in response to DNA damage underlie the brain region selectivity observed in neurodegenerative diseases.

## DNA Polymerases that are Active in Neurons in Response to DNA Damage

Humans encode 15 DNA polymerases belonging to the A, B, X, and Y families. Among these polymerases, Polα, Polδ, and Polε are the members of the B-family that carry out the bulk of DNA synthesis, whereas Y family members, Polη, Polι, Polκ, and Rev1, together with Polζ, are mainly responsible for translesion DNA synthesis (TLS). The X family DNA Polβ, λ, μ, and terminal transferase TdT are involved primarily in DNA repair, but Polβ, λ, μ can also partake in TLS. Humans possess three A-family DNA polymerases: Polγ, Polθ, and Polν. Polγ is a high-fidelity mitochondrial polymerase responsible for the replication and repair of mitochondrial DNA. By contrast, Polθ and Polν are low-fidelity nuclear polymerases that participate in TLS and DNA repair. PrimPol is the latest entrant, a member of the archaeo-eukaryotic primase family of enzymes, which brings the total number of bona fide DNA polymerases in humans to 16. Each polymerase has distinct features in DNA synthesis and distributes differently in tissues and cell cycle phases. These features will determine their performance and position in diverse DNA repair pathways.

### DNA Polβ Mediates the Short Patch Base Excision Repair in Differentiated Cells, Whereas DNA Polβ Together With δ/ε is Responsible for the Long Patch Base Excision Repair in Proliferation Cells

Base excision DNA repair (BER) is proposed as the main DNA repair pathway in mammalian postmitotic neuronal cells in that it processes the majority of smaller lesions that do not greatly distort the helical structure of DNA. BER targets many of those due to oxidative damage, a major threat in aerobic organisms; N-alkylated bases generated from environmental agents in the processes of metabolism; and the DNA lesions that result from hydrolytic reactions that occur indiscriminately in all organisms (e.g., resulting in abasic sites come from depurination). Most well-known among these are the highly mutagenic 8-oxoG and CydA adenine, but none constitutes more than a few percent of the total oxidative damage.

**Figure 1:**
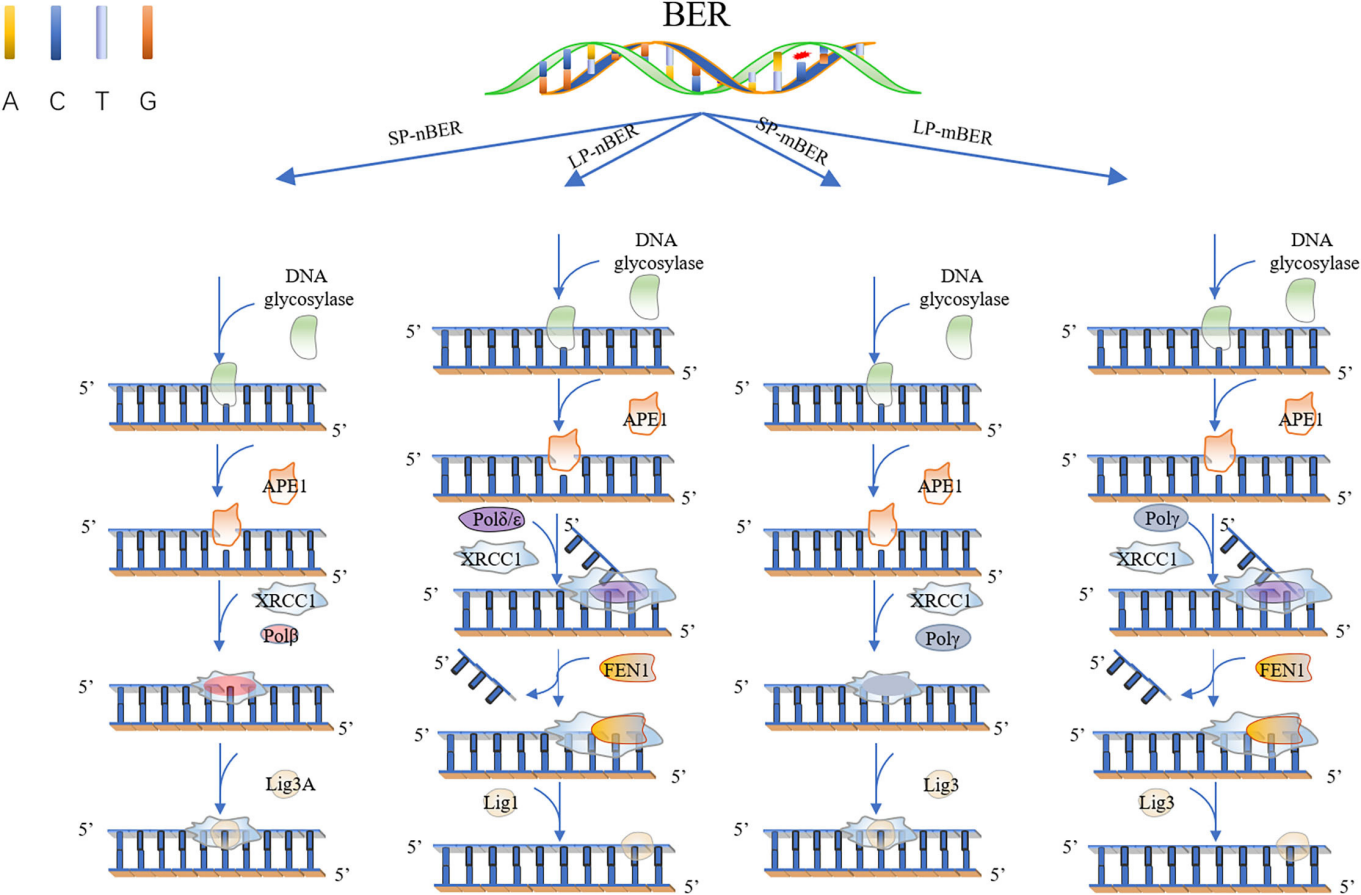
Base excision repair. In short patch nuclear BER (SP-nBER), DNA glycosylase can cut the chemical bond between nucleotide bases and ribose to release a complete DNA phosphoribose chain. This process will form an purine or pyrimidine (AP) site. APE1 cleaves the 5'position of the phosphodiester chain at the AP site. In this way, a 3'-hydroxyl group and a 5'-basic deoxyribose phosphate group appear on the DNA strand. When 3'-hydroxyl and 5'-deoxyribose phosphate (dRP) are present, the SP-BER pathway continues, where DNA polymerase β (Polβ) removes 5'-dRP and inserts a new nucleotide to fill the gap, and then X-ray repairs the complex of cross-complementary protein 1 (XRCC1) and DNA ligase 3 (LIG3) to close the cut. In long patch nuclear BER (LP-nBER), the 5' end is not a Pol β substrate. In this pathway, 2–10 nucleotides at the 3'-end are replaced and removed from the DNA backbone, and the new nucleotide chain and petals shape endonuclease 1 (FEN1) complexes, and base complementary pairing is performed under the action of POL (δ or ε). The final ligation step is performed by LIG1. Mitochondrial BER (mtBER) includes short patch BER (SP-mBER) and long patch BER (LP-mBER). SP-mBER is initiated by a specific DNA glycosylase, which recognizes modified or inappropriate bases and cleaves N-glycosidic bonds to produce abasic site. The resulting AP site is processed by AP endonuclease, resulting in a strand break with a 3'-hydroxyl end and a 5'-(dRP) residue. Then, the mitochondrial DNA polymerase pol γ fills in the single-nucleotide gap for repair. In addition to polymerase activity, pol γ has 3'−5' exonuclease and 5'dRP lyase activities. Therefore, when mtBER is initiated by a monofunctional DNA glycosylase, the 5'-dRP part produced when the AP endonuclease cleaves the strand can be removed by the 5'-dRP lyase function of pol γ. Finally, the resulting nick is sealed by DNA ligase. mtBER can also be performed LP-mBER, which involves the incorporation of 2–12 nucleotides during the repair synthesis process. The LP-mBER treatment of DNA damage causes the DNA strands to be exposed as a part of a single-stranded overhang or flap structure. These flap structures are recognized and cleaved by flap endonuclease 1 (FEN-1), which is an essential enzyme for nuclear LP-mBER, and then ligated by DNA ligase.

There exist two major BER pathways, which target different 8-oxoG mispairs, namely, “short-patch” pathway (SP-BER) for a single-nucleotide repair and “long-patch” pathway (LP-BER), as shown in Figure 1, which replaces a stretch of 2–12 nucleotides starting at the damaged site.

For a classical SP-BER, the pathway is initiated by a lesion-specific DNA glycosylase, e.g., the OGG1, removing 8-oxoG in C:8-oxo-G base pair, followed by AP endonuclease cleavage of the phosphodiester bonds, therefore producing a nick with 5′-deoxyribose-5-phosphate (5'-dRP). After sugar removal, the repair intermediate consists of a single-nucleotide gap that must be processed to have a 3′-hydroxyl and 5′-phosphate, usually by APE1 in mammalian cell, but not exclusively, by other enzymes, such as TDP1 and PNKP polynucleotide kinase phosphatase. At this point, classical BER converges with repair of single-strand breaks (SSBs), which is another type of lesion very frequently formed by ROS. SSBs are discontinuities in one strand of the DNA double helix, usually accompanied by loss of a single-nucleotide and holding chemically modified DNA termini near the break. In the next step of classical SP-BER, Polβ, together with X-ray repair cross-complementing protein 1 (XRCC1) and DNA ligase IIIa (Lig IIIa), forms the core BER complex and engages into the lesion sites. Polβ is composed of two specialized domains. The smaller N-terminal domain (8 kDa) processes 5′-deoxyribose phosphate (dRP) lyase activity, whereas the C-terminal domain (31 kDa) has the polymerase activity which is responsible for DNA synthesis. Based on these activities, Polβ replaces the missing nucleotide and catalyzes the removal of the 5'-dRP moiety to generate a normal 5'-phospho-nucleotide, which can be ligated to the 3'-hydroxyl in the final BER step (Kim and Wilson, 2012). The activity in excision of 5′-dRp seems to be more important to Polβ in SP-BER, for exogenous expression of the separated N-terminal lyase domain, but not the polymerase domain, induced resistance to monofunctional alkylating agents in Polβ knockout cells. Notably, the lyase activity is problematic for processing certain oxidized APs, as Polι and λ, which carry out alternative BER, also have 5′-dRP lyase activity.

**Figure 2:**
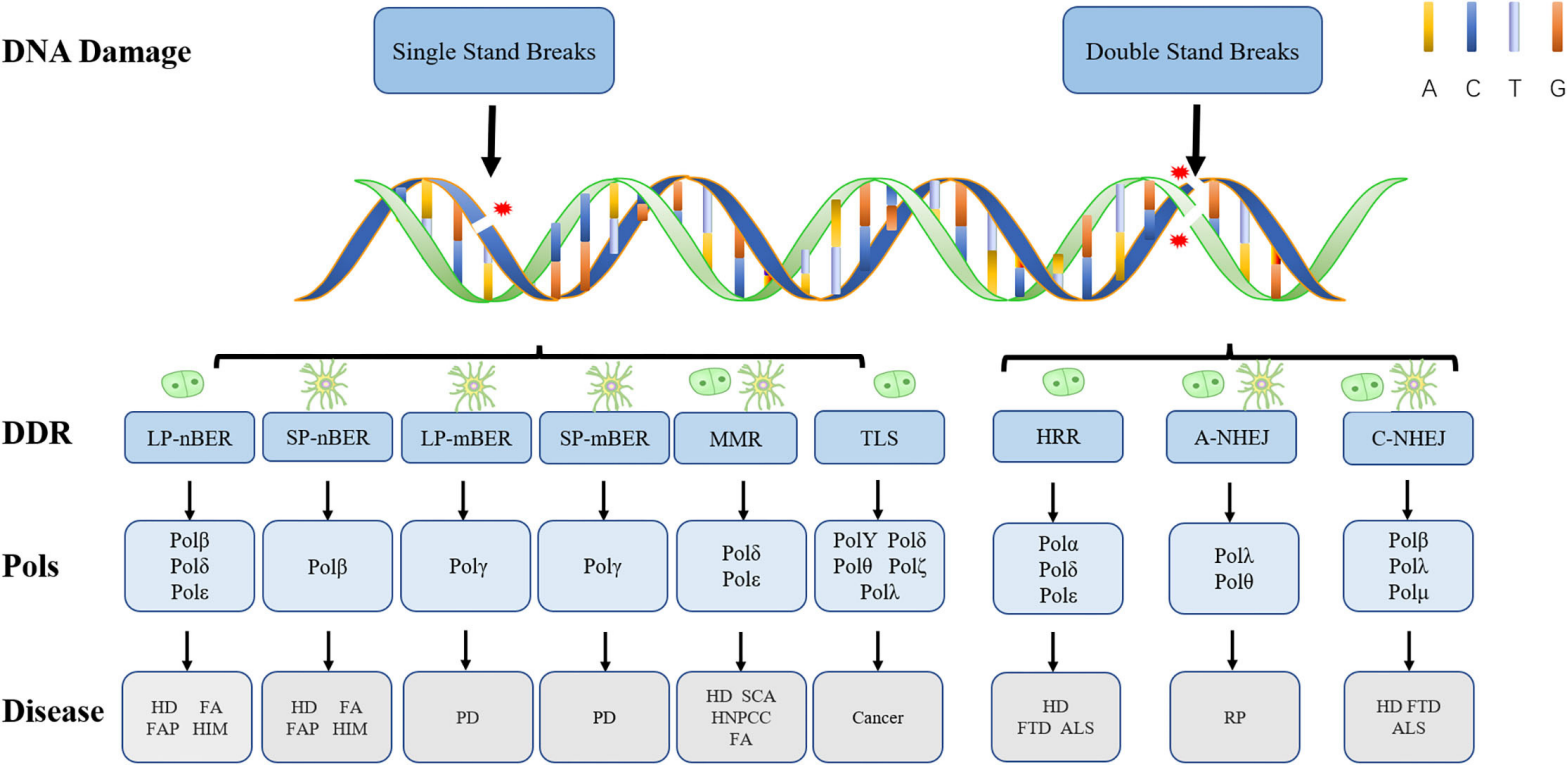
Types of DNA damage and corresponding repair pathways. Endogenous and exogenous factors can induce different types of DNA damage. Here, we show their DDR mechanisms and cell proliferation status. The nerve cell model in the figure represents non-dividing cells, and the other model represents dividing cells. While LP-nBER, TLS and HRR exist in dividing cells, SP-nBER, LP-mBER, and SP-mBER exist in non-dividing cells. By contrast, MMR, A-NHEJ, and C-NHEJ exist not only in non-dividing cells but also in dividing cells. Here also shows DNA Pols related to different DNA damage repair pathways and diseases related to different types of damage. It is worth noting that Polγ only plays a role in mtBER. ALS, amyotrophic lateral sclerosis; FA, Fanconi syndrome; FAP, familial adenomatous polyposis; FTD, frontotemporal dementia; HD, Huntington's disease; HIM, hyper-IgM syndrome; HNPCC, hereditary non-polyposis colorectal cancer; PD, Parkinson's disease; SCA, spinocerebellar ataxia.

In other cases, when a 5′-sugar phosphate group is modified (oxidated or reduced) and resistant to the Polβ dRP lyase activity, the removal of this sugar phosphate will occur by the “hit-and-run” mechanism, the pathway will switch to LP-BER, as shown in Figure 1. LP-BER involves PCNA and RFC, which load one of several polymerases sequentially, including Polβ, Polδ/ε that synthesize at least two nucleotides, displacing the downstream DNA molecule. Polβ may also initiate long-patch repair of modified 5′-dRP by inserting the first nucleotide (Dyrkheeva and Lavrik, 2021) (Figures 1, 2). The resulting 5′ flap is finally removed by FEN1 and ligated with Lig I.

It seems redundant that so many polymerases are recruited to carry out one task, but all the events may be relevant to the low-fidelity of Polβ.

Polβ is an X family polymerase. It lacks an intrinsic 3′ to 5′ proofreading exonuclease activity that enhances the accuracy of other DNA polymerases and shows an average error rate of approximately 1 per 4,000 nucleotides inserted when extending 5′-single-strand regions or gaps (Brown et al., 2011). Correspondingly, the overexpression of Polβ in cells suggested a Polβ dose-dependent mutator phenotype (Canitrot et al., 1998), and cancer cells with increased expression of Polβ showed elevated level of frameshift errors during BER (Azambuja et al., 2018). From this point of view, it seems incredible that base modifications are restored at the cost of increase in mutation rate, especially in non-dividing cells that base modification repair is not a burning question.

Actually, cells resolve the problem in two ways: the fidelity of Polβ increases up to 100-folds when it catalyzes the filling of 1-nt gap with a 5′-phosphate moiety, as what it is doing in SP-BER, compared with substrates containing mononucleotide gap without 5'- phosphate or a >1 nt gap with a 5′-phosphate group (Osheroff et al., 1999), this property of the enzyme largely reduces the mutagenicity of SP-BER; Another way is the LP-BER. In LP-BER, Polβ and Polδ/ε work in a highly sequential manner. Polβ possesses a PCNA-interacting protein (PIP) motif and can interact with PCNA, a component of the LP-BER, as well as an essential property for polymerases to participate in polymerase switching. PCNA mediated the switch from Polβ to Polδ/ε. Polβ participates in LP-BER particularly in the early stage, whereas DNA Polδ and Polε can mediate the following gap filling in concert with PCNA. A recent study provides evidence that in the presence of PCNA, the processivities of Polε and Polδ are actually quite similar (reviewed in Abbotts and Wilson, 2017). Generally, Polδ was defined as the lagging strand polymerase, whereas Polε as a component of the leading strand replisome (Burgers and Kunkel, 2017; Li and O'Donnell, 2018). But Polε and Polδ are the only two eukaryotic nuclear DNA polymerases with an intrinsic 3′-5′ exonucleolytic activity with which to excise primer terminal nucleotides. This activity is especially useful for correcting the proofreading errors made by Polβ.

Accordingly, Polβ is the predominant enzyme in both neurons and astroglia cells of rat cerebral cortex at all the postnatal ages, whereas relative abundance of DNA polymerases other than Polβ is the higher percentage of Polδ/ε in both cell types. A notable difference in neurons and astroglia cells regarding the relative abundance of DNA polymerases other than Polβ is the higher percentage of Polδ/ε in neurons and a more sustained Polα activity through the life span in astroglia. These results indicated the activity of short patch and long patch base excision repair in neurons. It is quite possible that Polβ and Pol δ/ε present in neuronal cells are aptly suited for the purpose of BERs.

### Polλ Mediated Non-canonical LP-BER—Exclusive for Mitotic Cells in Removing Oxidative Induced Mismatch Pairs

There also exists a non-canonical LP-BER, when the C:8-oxo-G base pairs escape from SP-BER. In this way, during replication, the DNA synthesis polymerase incorporates a wrong A opposite 8-oxo-G, giving rise to A:8-oxo-G mispairs, due to the identical conformation of 8-oxo-G and a thymine. MUTYH, another DNA glycosylase, recognizes A:8-oxo-G and initiates the non-canonical LP-BER (van Loon and Hübscher, 2009; Trasviña-Arenas et al., 2019). MUTYH catalyzes the excision of the wrong A from opposite 8-oxo-G, leading to the formation of an AP site (Markkanen et al., 2013). This AP site is further processed by APE1, resulting in a 1 nt gap with 3′OH and 5′dRP moieties. Subsequently, Polλ with the help of the cofactors PCNA and RP-A incorporates the correct C opposite 8-oxo-G to achieve the strand displacement (Trasviña-Arenas et al., 2019). Finally, FEN1 cleaves the 5′ flap to generate a 5′-P group, which could be ligated by DNA ligase I to yield an intact C:8-oxo-G containing dsDNA. Thus far, the C:8-oxo-G is then again the substrate for SP-BER subpathway.

Compared to C:8-oxo-G, A:8-oxo-G mispairs are coupled with replication, that is to say, A:8-oxo-G base pairs emerge only in S/G2 phase. It is interesting that Polλ, the key enzyme of non-canonical LP-BER, experiences an S-phase recruitment to chromatin, which was even increased upon oxidative stress. The synchronously response of Polλ indicates that non-canonical LP-BER is in place to guarantee the availability of productive repair complexes at the exact time that A:8-oxo-G mismatches are being produced during replication. Notably, like its “partner” MUTYH, Polλ seems to be primarily needed on chromatin during S-phase, whereas its counterpart in classical BER Pol β is considered more of a housekeeping enzyme active all through the cell cycle.

As a translesion synthesis polymerase with low fidelity, it is surprising that Polλ is responsible for inserting the correct “C” opposite 8-oxo-G, as most polymerases show significant error-prone bypass of 8-oxo-G. In fact, Polλ has a unique ability to insert 1,200-folds more correct C opposite 8-oxo-G than incorrect A with the help of PCNA and RPA (Burak et al., 2016). The auxiliary proteins PCNA and RPA are more likely to act as molecular switches in this context to activate the error-free Polλ at the same time repressing error-prone bypass by the canonical BER enzyme Pol β (Maga et al., 2008; Belousova and Lavrik, 2015). Though PCNA is non-cell cycle regulated, RPA, as a typical replication-associated factor, is most frequently upregulated during S phase (Hagen et al., 2008).

### Polγ–Mitochondria Base Excision Repair

DNA repair in mitochondria is mostly limited to base excision repair (BER), which is the best characterized DNA repair process among mtDNA repair mechanisms. Identical to nuclear BER (nBER), mitochondria BER (mBER) can also proceed *via* SP-BER (SP-mBER) and LP-BER (LP-mBER) (Figures 1, 2) (Copeland and Longley, 2008). Both of these pathways are initiated from the cleavage of an oxidized or damaged base by a specific glycosylase, leaving an abasic site that is cleaved on the 5′ end by AP endonuclease (APE) to generate a nick with a 5' dRP flap. During SP-mBER, Pol γ fills the gap and cleaves the 5′-dRP moiety prior to ligation by ligase III. But when the 5′dRP group is oxidized, Polγ can proceed to fill the gap and displace the downstream DNA generating a flap structure. This flap structure can then be cleaved by either FEN1 or DNA2. Alternate to Polγ, Polβ has also been implicated in the mBER (Prasad et al., 2017; Sykora et al., 2017; Baptiste et al., 2021).

Though several mBER proteins share the gene code except splice differently with the nBER, e.g., OGG1 and uracil-DNA glycosylase (UDG) (Nilsen et al., 1997; Nishioka et al., 1999), Polγ is an mitochondrial polymerase responsible for repair and replication events in mitochondria (Baptiste et al., 2021). It is a family A DNA polymerase of very high fidelity due to high insertion discrimination and an intrinsic proofreading activity.

An age-dependent decline of mBER activities and a decrease in the expression of OGG1 and Polγ enzymes were observed in rat cerebral cortices (Chen et al., 2002), and it was also reported that there was an age-dependent decrease of mtDNA glycosylases activities in five different mouse brain regions (Imam et al., 2006). Homozygous Polγ knockout mice are inviable *in utero* due to an early developmental arrest (Hance et al., 2005). Mutations in Polγ are reported to be associated with neurodegenerative diseases in which skeletal muscle and nervous tissues are most frequently affected, e.g., progressive external ophthalmoplegia and Alpers' syndrome (a progressive, neurodevelopmental, mitochondrial DNA depletion syndrome) (Hedberg-Oldfors et al., 2020) and case of progressive neuro-ophthalmic manifestation with optic atrophy, mixed polyneuropathy, spinal and cerebellar ataxia, and generalized chorea (Dosekova et al., 2020) (Figure 2). There is also evidence that mutations or alterations in expression in Polγ are associated with AD (Wallace, 2005) and PD (Kraytsberg et al., 2006).

### How Does the Cell Determine Which BER Subpathways Should Be Turned On?

It seems that BER is dependent on cellular proliferative status.

As C:8-oxo-G is formed whenever oxidative stress insults the C:G base pair, it should in principle be handled effectively by SP-BER, which is indeed predominant in differentiated cells. In line with the enrichment of C:8-oxo-G in non-dividing cells, Polβ is apparently the main DNA polymerase in rat brain (Rao, 1997). Furthermore, the activity of Polβ in extract from non-proliferating cells is ~2-folds higher, relative to extracts from proliferating cells (Akbari et al., 2009).

For the expression and activity of Polβ conform the cell cycle, which is high in G1 but low in S-G2/M, we speculated that Polβ mainly involved SP-BER may be more important for postmitotic cells to repair the multitude of frequent, constantly arising DNA base alterations and single-strand breaks (SSBs), prompting a comparison with the role of a cellular housekeeper engaged to keep genomic DNA tidy and clean. In addition, the major components of canonical BER, such as OGG1, APE1 and PNKP in SP-BER, and PCNA and FEN1 in LP-BER, are proved to be not cell cycle regulated (Akbari et al., 2009). The cell cycle-independent expression of these proteins facilitates the operation of BER in non-dividing cells. Nevertheless, LP-BER contributes more in proliferating cells rather than in neuron cells. The level of LP-BER proteins, including FEN1, PCNA, Polε, and LIG1, was observed to be decreased in neuron cells, resulting in an overall reduced LP-BER capacity after neuronal differentiation. The neuron has a significantly changed BER system compared to the neuroblast that relies heavily on Polβ with replicative polymerase δ/ε strongly attenuated. This may leave the neuron more vulnerable to certain forms of DNA damage preferentially repaired by LP-BER in replicating systems. Therefore, oxidative stress-induced DNA damage may be better tolerated in replicative cells than in non-proliferative cells due to a more robust LP-BER activity that can modulate repair of substrates more efficiently (Sykora et al., 2013).

### BER/SSBR Defects and Neurological Disease

**Figure 3:**
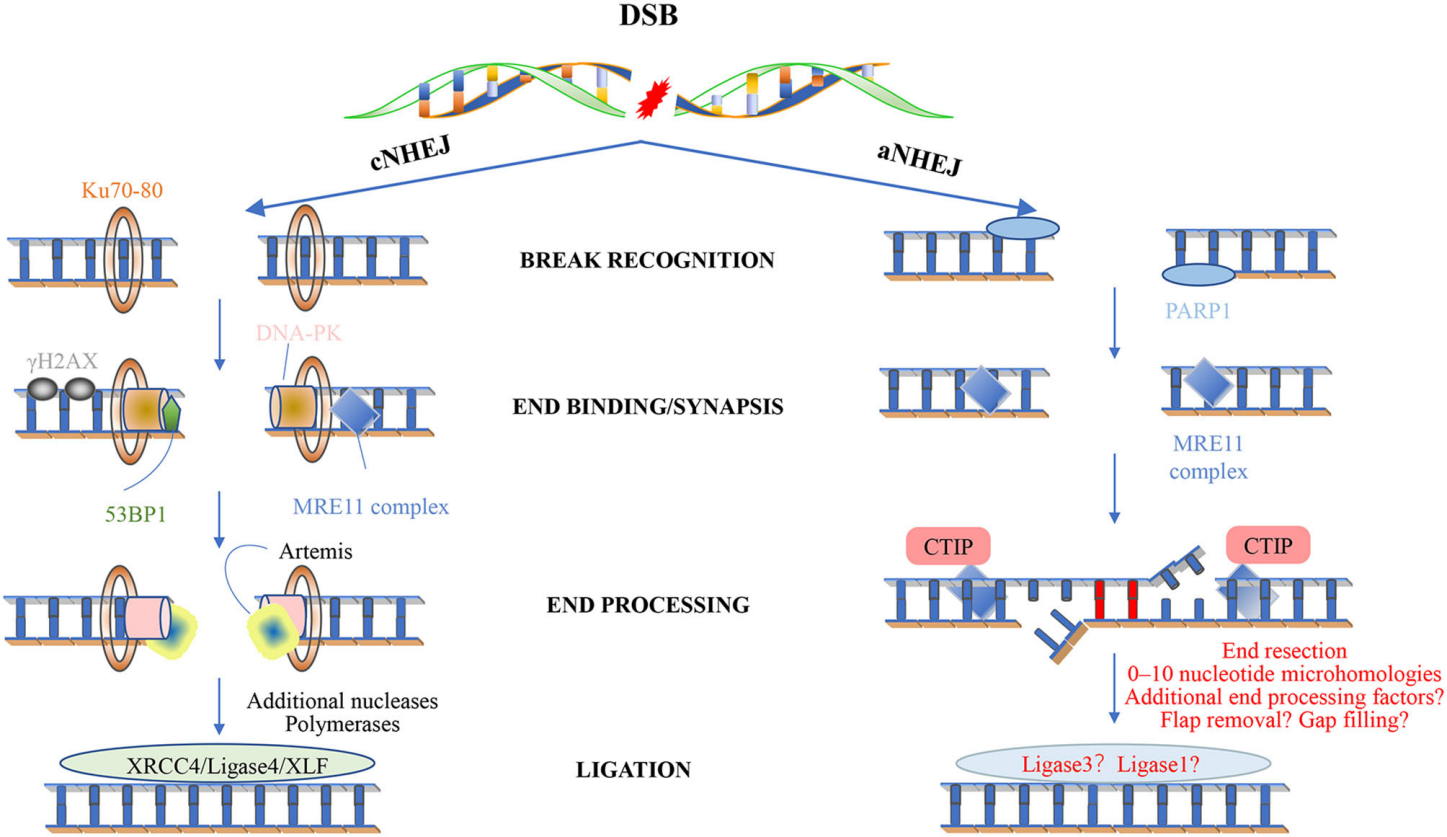
Non-homologous end joining. Classical NHEJ (cNHEJ) is triggered by the binding of a Ku heterodimer to the fragmented DNA end and provides a scaffold for the recruitment of other factors, including DNA-PKC, XRCC4 ligase IV-XLF, Artemis, and DNA polymerase. The Mre11 complex is loaded to the end of the DNA and can recruit ATM. The histone variant H2AX is phosphorylated to form γ-H2AX, which is located on both sides of the fracture. This in turn promotes the recruitment of other factors, leading to the assembly of large multi-protein complexes that may play a role in disrupting the signal, repairing and keeping DNA ends together, and minimizing the chance of abnormal rearrangements. cNHEJ requires additional enzymes to prepare the DNA-attached ends. One of the enzymes is Artemis, and once DNA-PK stimulates the endonuclease activity in Artemis, it will hit the development clamp. The last step of cNHEJ involves binding the DNA ends through the DNA ligase IV/XRCC4/XLF complex. In short, cNHEJ is a repair process in which the ends of DSBs are directly linked by DNA ligase, which does not rely on homologous DNA sequences. The Ku protein (Ku70/Ku80) complex recognizes and binds to the end of DSBs, and the Ku-DNA complex recruits DNA-dependent protein kinase catalytic subunits (DNA-PKcs) to activate its kinase activity, phosphorylate itself to initiate the NHEJ pathway, and attract the recombinase Artemis Join to process the DNA ends, and then summon the XRCC4-DNAligase4-XLF complex to promote the ligation of the DNA ends. Less is known about the mechanism of alternative NHEJ (aNHEJ). Although PARP1 can interact with free DNA ends, is associated with DNA damage induction, and can interact with ATM, the Mre11 complex seems to play an important role. However, the mechanism of its action is still unclear. Recent studies have shown that aNHEJ also occurs in cells that can activate other repair pathways. Future research will definitely learn more about this mechanism and its genome editing potential.

Since SP-BER exerts pivotal repair activity to restore oxidative and alkylated bases in neurons, components in SP-BER should be speculated to link with neuronal disorders. First of all, as we concerned about, Polβ is closely related to neurogenesis and degeneration. Loss of Polβ leads to neonatal lethality in mice due to failed neurogenesis (Onishi et al., 2017; Uyeda et al., 2020). As mentioned above, due to the high levels of oxygen consumption in the brain, increased oxidative stress has been reported to be a significant event in aging and Alzheimer's disease (AD) (Figure 3). In neurons, BER, the major repair pathway responding to oxidative DNA damage that is primarily dependent on Polβ, is reduced during aging and AD. Decreased levels of Polβ have been observed from the brain tissue of patients with AD (Copani et al., 2006), mild cognitive impairment (MCI) (Weissman et al., 2007), or down syndrome (Patterson and Cabelof, 2012), a genetic disorder that is reported to have an increased risk of developing AD. Later studies provide evidence that this reduction of Polβ in aging and AD can render neurons more vulnerable to dysfunction and death, causing neurodegeneration and exacerbated AD phenotypes (Sykora et al., 2015), impairing olfaction through endangering olfactory bulb neurons (Misiak et al., 2017). These studies support the notion that Polβ is involved in the pathogenesis of AD, and bolstering DNA repair through Polβ may protect neurons against dysfunction and degeneration in aging and AD.

Other core constituents of BER, such as APE1, XRCC1, and LIG3, are also incompatible with embryonic or postnatal survival (Brenerman et al., 2014). Brain-specific conditional knockout of LIG3 in a mouse model brought about develop retardation and severe ataxia-associated cerebellar neurodegeneration (Gao et al., 2011). Similarly, mice that are knockout of XRCC1 in brain exhibited rapidly progressive loss of cerebellar and hippocampal neurons during the postnatal period, associated with ataxia with life span for approximately 4 months (Lee et al., 2009).

Proteins involved in BER have been recognized to have linkages to neurological disease in humans (McKinnon, 2009; Rulten and Caldecott, 2013). Recently, multiple distinct human syndromes have been linked to inherited mutations in PNKP. These include microcephaly with seizures (MCSZ), which is characterized by microcephaly, early-onset, intractable seizures and developmental delay, progressive cerebellar atrophy and polyneuropathy (Shen et al., 2010; Carvill et al., 2013; Nakashima et al., 2014), and ataxia with oculomotor apraxia type 4 (AOA4), characterized by neurodegeneration (Bras et al., 2015). Supporting with that, mice harboring a brain-specific PNKP knockout exhibit cortical and cerebellar neuron loss and early postnatal death, whereas an MCMZ mouse model expressing an intermediate level of PNKP protein demonstrates generalized neurodevelopmental and maintenance defects, including microcephaly (Shimada et al., 2015). PNKP has also been connected to the pathology of SCA3, due to the inhibition of PNKP phosphatase activity by the repeat expansion present in SCA3 (Chatterjee et al., 2015; Gao et al., 2015; Chakraborty et al., 2020). Interestingly, TDP1, which executes similar activity to PNKP, are also involved in neuronal disorders. TDP1 is normally highly expressed in neuronal cells. The homozygous mutation in TDP1 (His493Arg) is reported to result in spinocerebellar ataxia with axonal neuropathy 1 (SCAN1) (Huang and Pommier, 2019). The diverse impact of BER/SSBR components mutations toward human disease provides important insights for understanding how genome stability pathways control tissue homeostasis. However, enhanced genome data analysis is still needed to fully comprehend the linkage of DNA repair-associated mutations and human disease.

### Polλ and Polμ Sew Broken Strands in Non-homolog End Joining Repair in Postmitotic Cells

Mammalian double-strand breaks (DSBs) can be repaired by homologous recombination (HR), “canonical” nonhomologous end-joining (C-NHEJ), “alternative” nonhomologous end-joining (A-EJ) (Figure 3), or by single strand annealing (SSA). Although kinetics and end structure are undoubtedly important in determining what pathway is used for DSB repair, it is clear that different cell types use different repair pathways at different rates. For example, HR appears to be especially efficient in stem cells, whereas NHEJ is used more frequently in more differentiated lineages. NHEJ is preferentially used by more differentiated lineages, not only for it acts the first attempt to restore the DSBs (Mao et al., 2008) and accounts for about 80% of the repair events (Beucher et al., 2009), but also for that it requires minimal homology for the repair of DSBs and operates throughout the cell cycle (G1 < S < G2/M) (Mujoo et al., 2017), whereas its counterpart HR operates majorly during S and G2 phases when a sister chromatid is available as a template (Du et al., 2018).

Nonhomologous end joining involves mainly four steps. Upon DSB induction, the Ku heterodimer binds to the DNA ends and protects them from further resection. DNA-PKcs is recruited and phosphorylates itself, as well as Artemis, which is an exonuclease. The activated Artemis processes the DNA ends, making them ready for end filling. Pol X family polymerases carry out the end filling. The final nick is sealed by the DNA ligase IV–XRCC4–XLF complex, XRCC4 and XLF, forming long filaments and helping to hold the DNA ends together.

A total of three of the four X-family DNA polymerases are implicated in DSB processing associated with NHEJ, namely, Polλ, Polμ, and the terminal deoxynucleotidyl transferase (TdT) (Figure 3) (Ramsden and Asagoshi, 2012). Whereas, the two former enzymes are ubiquitously expressed, expression of TdT is restricted to early developing lymphocytes, which we would not describe here (Loc'h and Delarue, 2018). All three polymerases possess an N-terminal BRCT domain, after recruitment, polymerase λ/μ can perform template-dependent, as well as template-independent DNA synthesis. Polλ acts as a backup function in non-classical BER, and as we have known, Polλ is far more prone to frameshift error. Why the cell uses it other than other polymerases with higher fidelity now is still an open question. Polμ relies on at least one paired base between the primer terminus and the template to remain active during *in vitro* NHEJ. With the NHEJ core factors Ku and XRCC4-ligase IV and take advantage of their end aligning activity, Polμ can promote NHEJ of noncomplementary ends. Polμ extends unpaired primer termini by template-independent addition (Kaminski et al., 2020).

Unrepaired or incorrect repair of DSB can lead to apoptosis or cancer. Failure to maintain genome stability is the cause of the decline of each organism through physiological and pathological processes, such as aging, neurodegeneration, and cancer (Loshchenova et al., 2020). HR leads to accurate repair, whereas NHEJ is mutagenic in nature. In actively circulating cells, the efficiency of NHEJ of compatible ends (NHEJ-C) is two times that of NHEJ of incompatible ends (NHEJ-I), and the efficiency of NHEJ-I is three times that of HR. Studies have shown that NHEJ is a faster and more effective way to repair DSB than HR. In quiescent or differentiated cells where G1 is arrested, the frequency of HR may be much lower. The ratio between NHEJ and HR varies greatly between phylogenetic groups. Indirect evidence for the role of HR in repairing ionizing radiation (IR)-induced DSB comes from the increased sensitivity of HR-deficient cells in the S and G2 phases of the cell cycle. However, mutants of the NHEJ pathway are very sensitive throughout the cell cycle and exhibit serious defects in repairing IR-induced DSBs (Beucher et al., 2009). In mammals and plants, NHEJ is the preferred route. The choice may be determined by the composition of the genome. Mammalian cells can avoid large genome rearrangements and accumulate deletions and insertions that lead to senescence and tumorigenesis.

The role of Polμ in the central nervous system has been recently studied. Increased neuronal death, disturbed axonal growth, and navigation were observed in the retinal ganglion cells (RGCs) of Polμ-deficient mice, providing new clues to the possible functional impact of impaired NHEJ pathway in the proper generation and the connectivity of neurons (Baleriola et al., 2016). Surprisingly, old Polμ-deficient mice were reported to have improved brain function, showing increased learning and brain long-term potentiation, which was possibly due to the delayed brain aging (Lucas et al., 2013).

## RNA Polymerase Mediated Transcription-Coupled DNA Repair

DNA lesions not only strongly impede replication, but also constitute barriers to the translocation of RNA polymerases (RNA pol) on the DNA template (Heckmann et al., 2019). While some DNA lesions cause transient transcriptional pausing, bulky DNA damage can cause prolonged transcriptional pausing and arrest, which signals for transcription-coupled repair (Crossley et al., 2019). As a result, the subsequent events involve transcription lesion bypass, DNA damage removal, and the changes in gene expression, leading to combined impact on transcription accuracy and efficiency, termed as “transcription stress” (Lans et al., 2019). The “transcription stress” would alter the profile of vital mRNAs, produce mutant transcripts, and worse increase genome instability, which may result in cellular dysfunction, senescence, or even premature cell death, all majorly contributing to aging (Lopez-Otin et al., 2013).

Here, we discuss below how RNA pol, which is involved in all the transcription-coupled responses to DNA lesion, coordinates with their partners to recognize the damage structure, to bypass the lesion or to recruit the repair proteins, and finally restarts the transcription cycle. In addition, we also try to elucidate the crucial link between RNA pol deficiency and the related human neuronal disorders.

### RNA Polymerase II—The Sensor and Scaffold of Transcription-Coupled DNA Damage

Among the three types of RNA polymerases in eukaryotes, RNA polymerase II (Pol II) is a key enzyme complex for the transcription of protein-coding genes, as well as non-coding RNA for synthesis (Lee et al., 2004). Ever since the determination of the complete 12-subunit (from RPB1 to RPB12) Pol II, rich and varied Pol II complex associated with a wide range of transcription factors, as well as DNA-binding molecules, lesions, and modifications, have been reported (Bernecky et al., 2016; Xu et al., 2016; Xu J. et al., 2017; Wang et al., 2018a).

In a simplified model of the transcription cycle, Pol II with general transcription factors is assembled on promoters to form transcription pre-initiation complexes (PICs), beginning the transcription (initiation). Pol II is highly processive, as well as dynamic that the Pol II complex (RNAP) continues along the strand of the targeted gene (elongation), yet pauses or even stops transcription once encountering signals, for example, collisions with other DNA-associated machineries or DNA lesions termed transcription-blocking lesions (TBL), that mark the transcription termination (termination).

Blockage of the elongating RNAP at the damaged site is the general trigger for transcription-coupled responses (Chen et al., 2018; Slyskova et al., 2018; Konovalov et al., 2019). As prolonged stalling of RNAP is detrimental to the genome stability and maintenance, it is important that the path of the elongating RNAP be cleared of obstructions. Here, we will ask how does Pol II distinguish the DNA lesion from normal terminal signal, and how does it determine which types of lesions it could bypass while others call for a transcription arrest? The answer is that depends on the lesion types.

We listed lesions that are originated from the insultation of metabolic intermediates and exogenous stimuli, which trigger different responses of RNA Pol II (Table 1).

Interestingly, Pol II responses diversely to the lesions, depending among other factors, on the “bulkiness” of the lesion. Non-bulky single-base modifications, such as alkylation and oxidized nucleotides, from normal endogenous cellular processes are very abundant, yet do not block Pol II. That is to say, these lesions would not be recognized by Pol II and be bypassed by the transcription machinery without initiating the transcription-coupled repair. While moderate helical distortions, especially UV-induced TT cyclobutene pyrimidine dimers (CPDs), can induce stalling due to nucleotide misincorporation opposite the lesion, followed by “error-free/prone” bypass, as observed for the CPD. In addition, strong helix-distorting DNA damages and bulky adducts generally cause steric blocks that prevent the entry of the damaged base into the active site of Pol II, and consequently a complete stalling of Pol II (Glatt et al., 2014; Patel et al., 2020).

### RNA Pol II Supports the Bypass of Non-bulky Lesions With a Similar Manner of Translesion Synthesis (TLS)—Big Threat to Neurons in Brain

**Figure 4:**
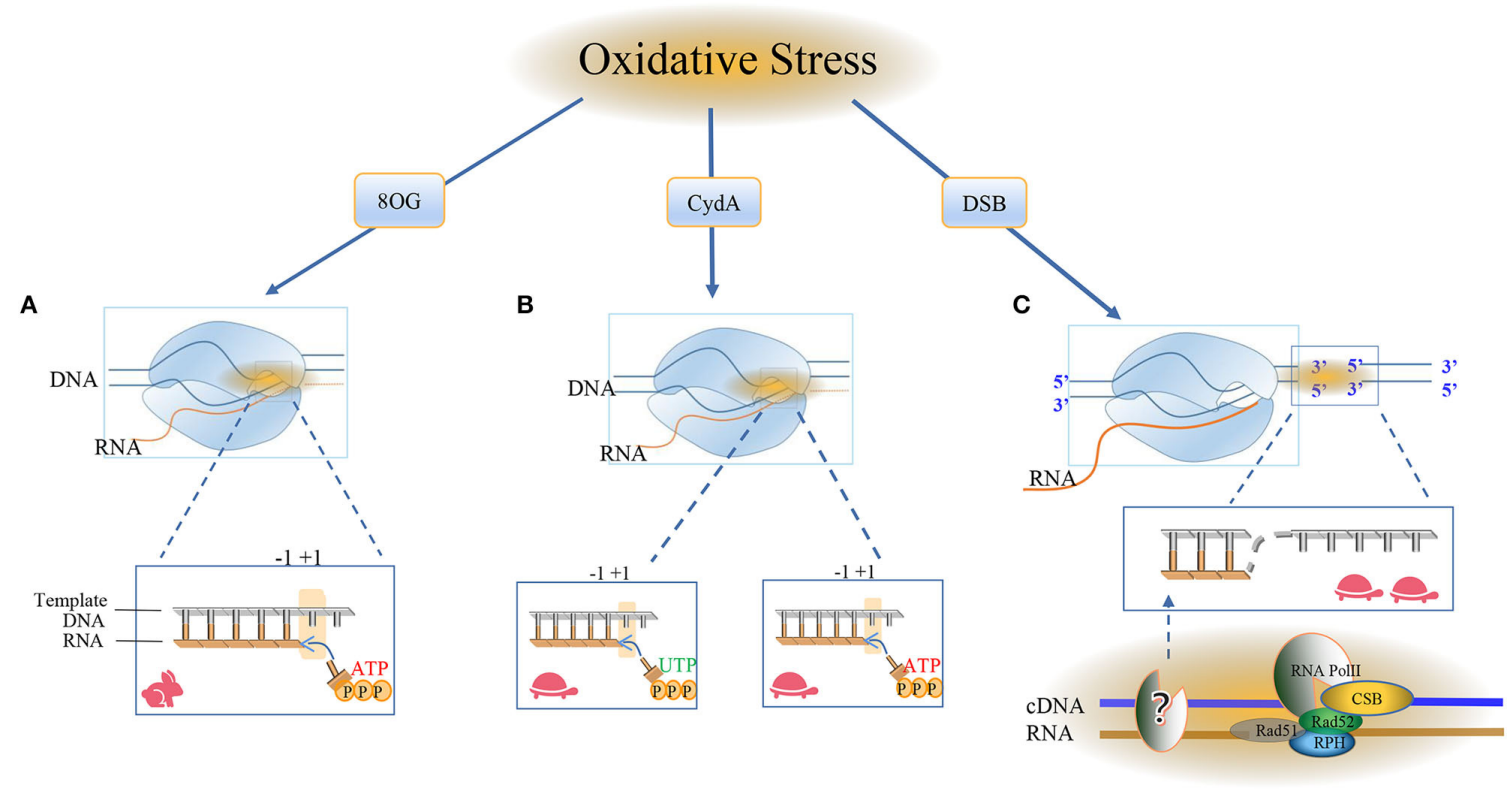
RNA Pol II bypasses the oxidative damage nucleotide addition cycle. The damage caused by oxidation here includes 8oxoG, CydA, and DSBs, and it is obvious that different cell types use different repair pathways at different rates. **(A)** Most of the oxidative modifications on bases can be bypassed by RNA Pol II. RNA Pol II bypasses the repair mechanism of oxidative damage and incorporates nucleotides into RNA. The RNA and Pol II form a ternary complex, in which the template DNA and the newly-born RNA form the core transcription bubble of Pol II. During the transcript extension process, RNA synthesis and the forward movement of Pol II will pass through the “Brown ratchet” coupling of translocation mechanisms. OG is one of the markers of oxidative stress. Pol II turns on the rapid transcription bypass, and DNA glycosylase triggers the release of specific DNA enzymes to identify and ablate damage and is used by BER in neuronal cells. Repair, the formation of ATP ready to pair with the base in the active site, the translocation mechanism creates a new RNA 3'end in the free +1 position in the active template, and RNA Pol II occupies the polymerase active site. **(B)** CydA is the damage in CydU that can cause Pol II transcription stagnation in human cells, opening a slow damage bypass, and polymerase is added to the UTP downstream of the CydA lesion and the DNA is pushed into the active position of RNA polymerase. Incorrectly adding AMP residue on the opposite side of the base of the downstream template, Pol II will translocate at the +1 position and slow down the subsequent elongation. **(C)** The surge of oxidative DNA damage puts too much pressure on the BER system and leads to DSB. Actively transcribed genes use transcription-coupled homologous recombination (TC-HR). RNA Pol II stagnates in the lesion, and the DNA-RNA hybrid structure recruits CSB. Then, the interaction between RNA Pol II and CSB initiates TC-HR and provides a scaffold for HR factors, such as Rad 52 and Rad 51C, which directly interact with CSB. There is a DNA polymerase upstream of the lesion site, which reverse-transcribes the template strand. The RNA polymerase II (Pol) in thermodynamics occupies the Pol active site. In the post-translocation state, elongation, nucleotide incorporation occurs through the “Bronen ratchet” site and reset Pol II to the pre-translocation state +1 (rabbit: fast, turtle: slow).

RNA Pol II evolves a distinct mechanism to bypass DNA lesions that the conformation of its active center is flexible enough to allow the accommodation of non-bulky modified bases, such as oxidative lesions (Walmacq et al., 2015). The moment it encounters base modification, Pol II switches transiently from a highly processive and error-free transcription mode to an error-prone mode, which is low NTP incorporation efficiency, finally generates different types of mutant RNA transcripts in a process called transcriptional mutagenesis (TM) (Walmacq et al., 2015). For example, Pol II supports the rapid transcription bypass of 8-oxo-guanine (8OG) lesion, which is the major DNA lesion resulting from oxidative stress, with both adenine misincorporation and correct cytosine insertions into the RNA strand (Konovalov et al., 2019). It is now clear that only the 8OG (syn) of the two 8OG conformation [8OG (syn) and 8OG (anti)] forms stable bp with the mismatched adenine in both the upstream position and the active position of Pol II, allowing the extension of the RNA strand (Figure 4A).

Contrast to 8OG, 8,5'-cyclo-2'-deoxyadenosine (CydA), another type of oxidative DNA lesion produced by hydroxy radical, strongly induces prolonged stalling of RNA Pol II, followed by slow transcriptional bypass, generating both error-free and mutant transcripts with AMP misincorporated immediately downstream from the lesion (Figure 4B).

**Figure 5:**
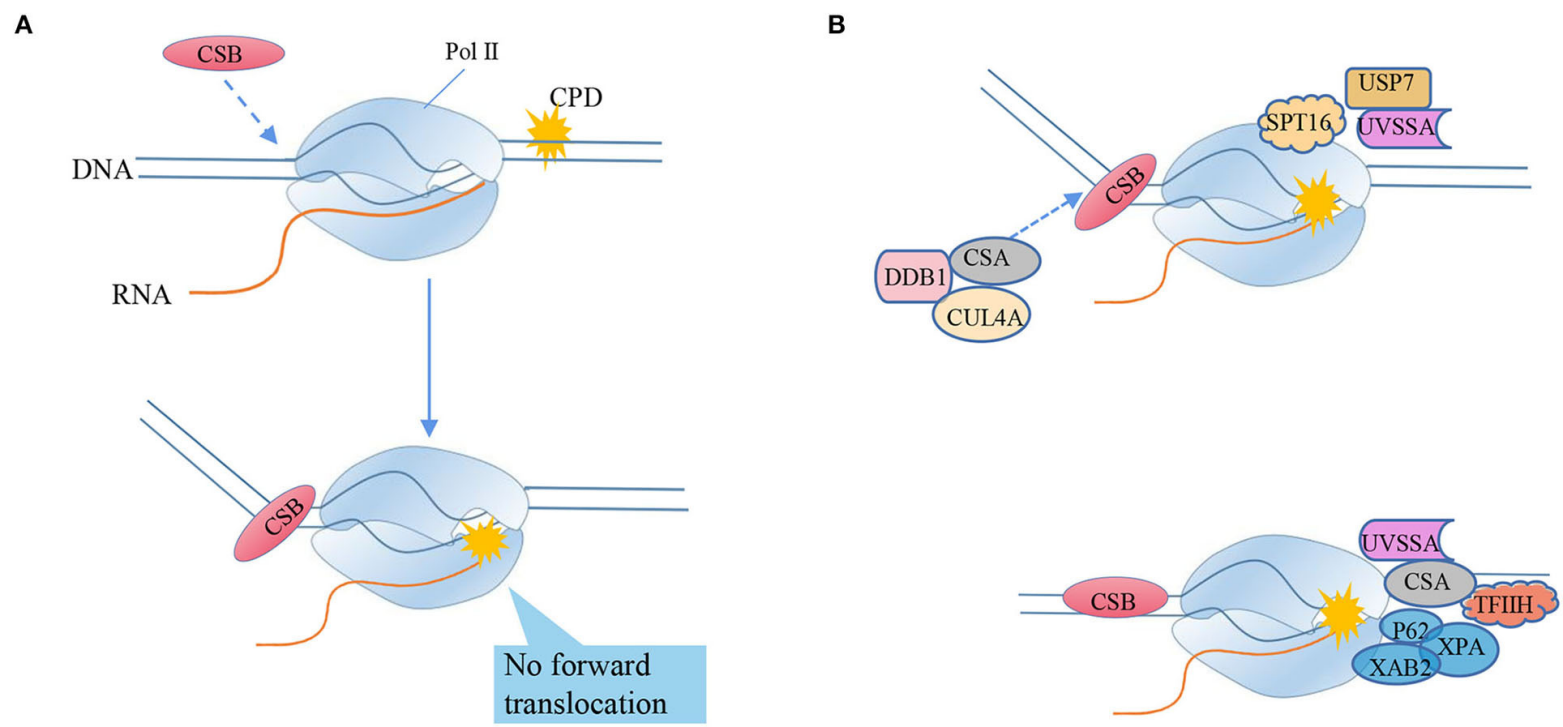
RNA polymerase II bypasses the transcriptional blocking damage cyclobutane pyrimidine dimer (CPD) triggers TC-NER repair. **(A)** CSB recognizes stagnant Pol II and binds to its upstream DNA, and uses its ATPase activity to push Pol II forward by translocating from 3'to 5'on the transcription template strand. CSB can move Pol II to natural pause sites and smaller lesions, but cannot push Pol II to larger TBLs, such as CPD (“stagnation”). The mechanism of detecting whether the captured Pol II can still translocate forward by pulling the DNA squeezed upstream can distinguish between the stagnant focus and the naturally suspended Pol II. **(B)** Transcription-coupled nucleotide excision repair model (TC-NER), from the arrest of RNA polymerase II (Pol II) to lesion excision and gap-filling DNA synthesis. After Pol II encounters TBL, its translocation activity induces the strong bending of upstream DNA and the tighter combination of CSB and Pol II, triggering transcription-coupled nucleotide excision repair (TC-NER). Chromatin remodeling agent stimulates the recruitment of CSB. CSA, DNA damage-binding protein 1 (DDB1) and cullin 4a (CUL4A)—RBX1 ubiquitin E3 ligase form the CRL4CSA complex, which is recruited to the lesion by CSB and recognizes damage signals after activation-NER starts. Ultraviolet-stimulated scaffold protein A (UVSSA) and ubiquitin C-terminal hydrolase 7 (USP7) are also recruited to the lesion, promoted by the chromatin remodeling subunit SPT16, and stably bind to CSA. CSB is ubiquitinated by CRL4CSA, but this is counteracted by USP7-mediated deubiquitylation to prevent CSB degradation. The transcription factor TFIIH is recruited through the interaction of its p62 subunit with UVSSA. TFIIH uses its 5'−3' XPD helicase to translocate forward on the DNA until it is blocked by the lesion, which may stimulate Pol II backtracking. XPA was confirmed to bind to TFIIH to recruit structure-specific endonucleases ERCC1-XPF and XPG. Cut the DNA 5' and 3'of the lesion, respectively, and release the 22–30 nucleotide long DNA oligomers containing the lesion. The resulting gap is filled by DNA synthesis, recruiting proliferating cell nuclear antigen (PCNA), replication factor C (RFC), and DNA polymerase δ, ε, and finally sealed by DNA ligase 1 or XRCC1-DNA ligase.

As a typical model of helix-distorting DNA damage, though CPD lesion is irrelevant with neurons, it is always adopted to study how polymerases react in response to bulky lesions. To our surprise, emerging evidence reported RNA Pol II slow bypass CPD with transient stalling in the lesion sites following a similar translesion model of CydA. Early studies using randomly damaged plasmids concluded that CPD lesions efficiently stall transcription, whereas several recent lines of evidence suggested that Pol II has an intrinsic capacity for bypass CPD with or without repair (Walmacq et al., 2012) (Figure 5A).

The bypass ability of RNA Pol II depends on the function of the subunit RPB1. RPB1 binds to nascent RNA and DNA, acts as a platform for modifications specifying the recruitment of factors that are responsible for lesion sensing and repair. Particularly, its two flexible regions participate in substrate binding, catalysis, and translocation. RPB1 not only accommodates 8OG, but also allows CPD lesions to enter the active site, covering the lesion with a 35-nucleotide footprint−10 nucleotides downstream and 25 nucleotides upstream of the lesion (Brueckner et al., 2007). The mutation variants RPB1-E1103G and T1095G, which promote the nucleotide insertion opposite to both thymines of the CPD, facilitate lesion bypass *in vitro* and increase UV resistance *in vivo*, whereas RPB1-G730D mutation, which abrogates bypass *in vitro*, consistently increases the UV sensitivity of RAD16-deficient yeast cells. Importantly, the increased UV resistance of the rpb1-E1103G mutant required the functional RAD26 gene involved in transcription-coupled-nucleotide excision repair (TC-NER) initiation, ultimately linking the translesion transcription to TC-NER. Thus, translesion transcription becomes essential for cell survival upon accumulation of the unrepaired CPD lesions in genomic DNA (Figure 5A).

Though the error prone bypass increases transcriptional errors and helps lesion escaping from Pol II transcriptional fidelity control checkpoints, as a matter of the fact, it is beneficial for somatic cells in that it supports rapid dividing by avoiding transcription cycle arrest while calls for alternative DNA repair machinery to resolve the DNA damage. However, the outcome of lesion bypass is more serious in brain cells. As the brain is thought to metabolize as much as a fifth of consumed oxygen, the reactive oxygen species (ROS) as the byproducts of metabolites consistently generate oxidative base modifications in brain. Though this type of DNA lesions is typically processed by the BER pathway, some lesions escape detection the chance of oxidative and pose a roadblock for transcription machineries in neuron cells. But notably, as long as lesions persist, mutant transcripts will accumulate and can influence cell function (Brégeon et al., 2009; Damsma and Cramer, 2009). Correspondingly, a recent study that stated administration of 8OG induces the accumulation of aggregable amyloid β peptides in cells expressing amyloid precursor protein, provided direct evidence in supporting this theory, and also established a link between Pol II-mediated transcription bypass and neurodegenerative diseases (Dai et al., 2018).

### RNA Pol II-Initiated TC-NER—When Stalled by Bulky Lesions

**Figure 6:**
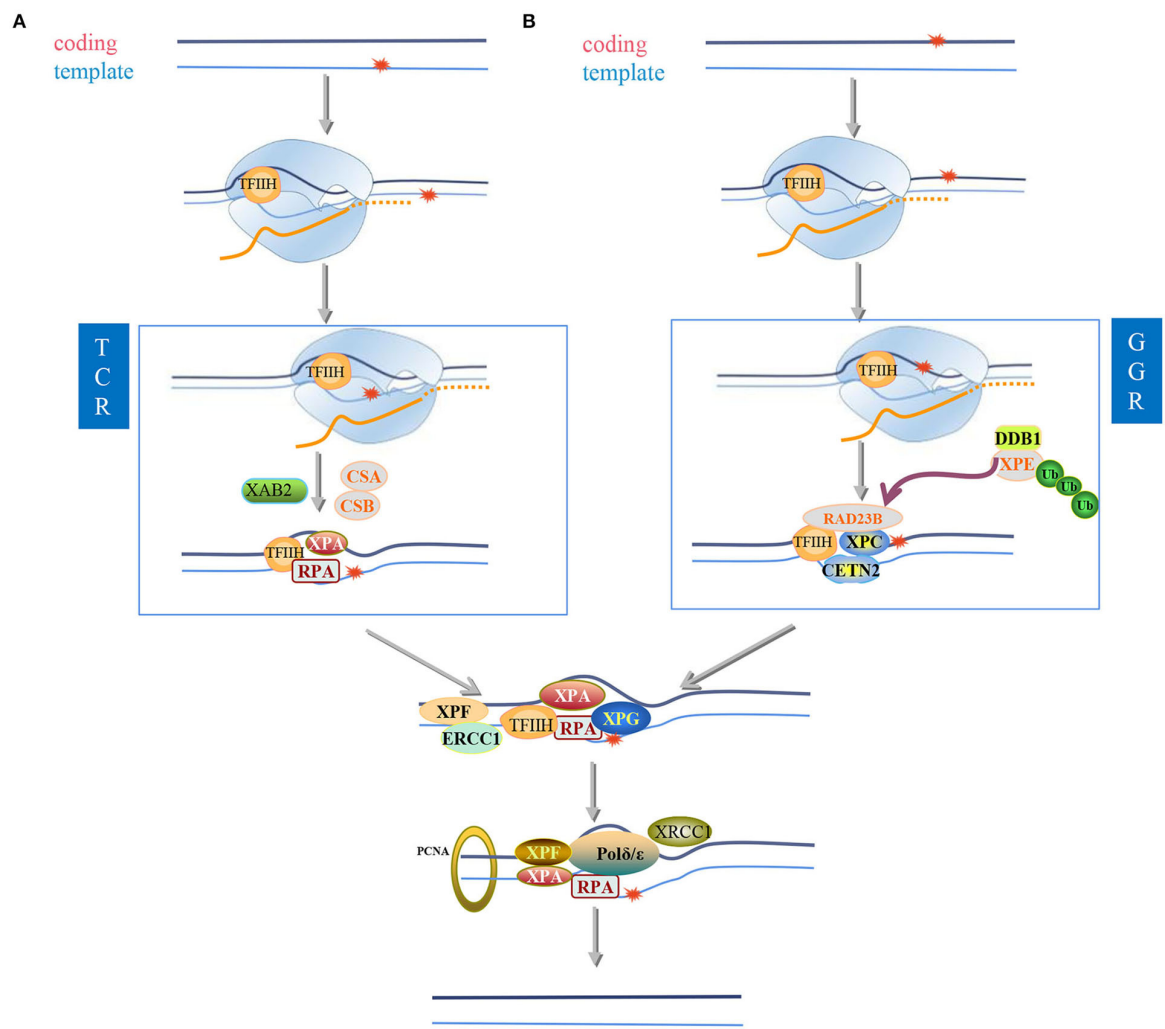
Nucleotide excision repair. In eukaryotic cells exposed to ultraviolet radiation, two different nucleotide excision repair (NER) modes are activated: (I) transcription-coupled nucleotide excision repair) (TC-NER) and (II) global genome Nucleotide excision repair (GG-NER), which participates in the recognition of twisted DNA and determines preferences related to space and time. NER helps eliminate spiral twisting damage, including cyclobutane-pyrimidine dimers (CPD), 6-4 photoproducts (6-4PPs), and other bulky adducts; therefore, it maintains the stability of the genome. The predictive influence of NER subpathway on the coding or template damaged chain of actively transcribed genes. **(A,B)** are located on the template strand **(A)** or coding strand **(B)** of the active gene to repair bulky lesions. If the lesion is located on the template strand **(A)**, it is read by RNAPII during the transcription process, and the lesion will cause the RNAPII complex to stall. CSA and CSB proteins are sensors of stalled RNAPII and recruit the transcription complex TFIIH to the lesion. The helicase activity XPB and XPD of the TFIIH complex open the chromatin around the lesion. XPA and RPA stabilize the open structure of chromatin. Endonuclease, ERCC1-XPF in 5'and XPG in 3'cut the damaged chain. Then, the gap is filled by DNA repair polymerase and ligase. If the lesion is located on the coding strand **(B)** and therefore cannot be read by the RNAPII complex, the lesion will not interfere with the synthesis of the enzyme and the gene will be transcribed. The lesion can be identified by the XPC complex in the future. TFIIH opens a denatured bubble of about 30 nucleotides around the lesion. The complex of XPC, RAD23B, and CETN2 can directly bind to the opposite DNA strand, where the spirally twisted lesion accumulates XPC is recruited to these damaged sites only after the UV-DDB (ultraviolet-damaged DNA-binding protein) complex binds. XPC complex is a disease-binding protein in global genome repair (GG-NER). After the DNA helix is partially opened, RPA (replication protein A) is added to the complex, which then helps in damage verification. XPA is best combined with single-stranded DNA (double-stranded DNA) structure, while RPA can only be observed in the ssDNA (single-stranded DNA region. This is the second NER subpathway. After the lesion recognition step, GG-NER and TC-NER are the same in the air bubbles before the incision, XPA has been shown to be located on the 5'side of the lesion. XPF-ERCC1 catalyzes the 5'incision, and XPG is responsible for the 3'incision around the lesion. ERCC1 is polyubiquitinated at the K33 site, which can be It is removed by USP45 (ubiquitin-specific peptidase 45). In TC-NER, when RNA polymerase II stays on the lesion during the transcription extension process, the lesion is recognized. RNA polymerase II (RNAPII) is due to active genes. The damage in the transcription chain (TS) stalls and attracts NER enzymes, RNAPII (RNA polymerase II), and CSB (Cockayne syndrome B protein) to further repair the protein. It can be deubiquitinated by USP7 to keep recruited in the lesion. Proliferating cell nuclear antigen (PCNA) is loaded to the 5'end of the DNA. PCNA interacts with XPA and XPF to stimulate their activity. The DNA region containing 22–30 nucleotides is excised from the complex DNA with TFIIH and then slowly released from TFIIH, bound by RPA or degraded by nuclease. During the nicking step, XPG is simultaneously ubiquitinated by CRL4Cdt2 and then degraded in the 26S proteasome. DNA synthesis is catalyzed by DNA polymerase δ/ε. Precise coordination of ubiquitin-mediated RNAPII removal after transcriptional blockade.

Nucleotide excision repair (NER) is arguably the most versatile pathway to restore bulky and helix-distorting DNA lesions, such as CPD, cisplatin-DNA crosslinks, and benzo[a]pyrene diol epoxide (BPDE)-DNA adducts (Kress et al., 2019; Gilbar and Pokharel, 2022; Martinez et al., 2022). It is an interesting mechanism that cells distinguish the DNA lesions on the template strand and coding strand and then repair them with two distinct subpathways of NER (Figure 6), namely, GG-NER and TC-NER. GG-NER is characterized by general patrol and repair lesions throughout the genome, including lesions on the coding strand of transcribed genes, whereas TC-NER only interfaces with RNA polymerase to repair lesions on the template strand of transcribed genes.

The major difference between GG-NER and TC-NER is the lesion recognition step. GG-NER utilizes XPC-Rad23B complexes to detect “helix-distorting” lesions, as shown in Figure 6B (Spivak, 2015). In contrast, TC-NER is initiated by the recognition of stalled Pol II by the main TC-NER factor Cockayne syndrome protein A and B (CSA and CSB) (Figure 6A). Since Pol II stalling also occurs at undamaged DNA, CSB, an ATP-dependent 3'-to-5' single-strand DNA translocase belonging to the SWI2/SNF2-family, is essential for cells to discriminate physiological pausing from transcription blocking lesions (TBL)-induced stalling (Krokidis et al., 2020; Tiwari et al., 2021).

The cryo-EM studies showed that Rad26, the yeast ortholog of CSB, binds to DNA upstream of Pol II and the TBL, which leads to an 80° bending of the extruding DNA. Notably, the translocase activity of Rad 26 pulls the DNA away from Pol II and therefore stimulates forward translocation of Pol II over the naturally occurring pause sites or small blocking lesions. But Rad26 fails to promote efficient transcriptional bypass of bulky DNA lesions that lead to strong blockage of translocation (such as CPD lesions). Notably, the binding of the HD2-1 “wedge” of CSB to Pol II in a region between the clamp (RPB2 side) and stalk (RPB4/7), antagonizes the repression of TC-NER by Spt5 and Spt4 on Pol II (Li et al., 2014; Duan et al., 2020) and facilitates the loading of downstream repair factors, such as UV-stimulated scaffold protein A (UVSSA), CSA, DNA excision repair protein ERCC-5 (XPG), and general transcription and DNA repair factor IIH helicase (TFIIH) on the CSB (Xu J. et al., 2017), leading to the initiation of TC-NER (Figure 5B). Subsequently, the core NER factors and several TC-NER-specific proteins, such as UVSSA, XPA-binding protein 2 (XAB2), TFIIH, and USP7 (Lake et al., 2010), are engaged. After that, the two subpathways of NER, GG-NER, and TC-NER, converge.

It is interesting to note that there is another distinct model that points out RNA Pol II, the subunit RPB9 of RNA Pol II to be exact, initiated the TC-NER independent of the yeast CSB homolog Rad26 (Li and Smerdon, 2002; Li et al., 2006). Loss of Rpb9 leads to genomic instability, aberrant segregation of chromosomes in mitosis in yeast and cells (Wery et al., 2004; Sein et al., 2018), and severely compromised the viability of Schizosaccharomyces pombe under genotoxic stress conditions (Bhardwaj et al., 2021). However, whether RPB9 induces neuronal dysfunctions *via* TC-NER is an open question to date.

### The Interplay of TC-NER and GG-NER in Postmitotic Cells—Linkage to Cockayne Syndrome of RNA Pol Function

Cockayne syndrome is a rare disorder characterized by cutaneous sensitivity to sunlight, abnormal and slow growth, cachectic dwarfism, progeroid appearance, progressive pigmentary retinopathy, and sensorineural deafness. There is delayed neural development and severe progressive neurologic degeneration resulting in mental retardation. Two clinical forms are recognized: in the classical form or Cockayne syndrome type 1, the symptoms are progressive and typically become apparent within the first few years or life; the less common Cockayne syndrome type 2 is characterized by more severe symptoms that manifest prenatally. Cockayne syndrome shows some overlaps with certain forms of xeroderma pigmentosum (XP). Unlike xeroderma pigmentosum, patients with Cockayne syndrome do not manifest increased freckling and other pigmentation abnormalities in the skin and have no significant increase in skin cancer.

Here, we want to discuss why CS mainly exhibits neurological pathologies, whereas patients with XP have a very high incidence of UV-induced skin cancer, though both share a common pathway NER? The answer may lay on the cell type-specific activity of TC-NER and GG-NER proteins.

Indeed, the recruitment and activity of NER endonuclease ERCC-1/XPF-1, which plays a pivotal role in damaged-strand incision during NER unhooking of inter-strand crosslinks and removal of DNA overhangs during DSB repair, is largely repressed in neuron cells in a C. elegans model (Sabatella et al., 2021). These data exemplify the importance of TC-NER rather than GG-NER for maintaining transcriptional integrity and cell functionality in postmitotic neurons, which likely correlates with the fact that neurodegeneration is a typical feature of human patients carrying mutations in TC-NER factors (Karikkineth et al., 2017; Lans et al., 2019). Though the function of TC-NER is greatly emphasized in neurons, GG-NER still works in the absence of TC-NER, and thus, GG-NER probably mainly functions to support the maintenance of transcribed genes as a backup system in neurons (Lans and Vermeulen, 2015; Sabatella et al., 2021). Such transcription-specific GG-NER activity has been previously dubbed “transcription domain-associated repair” in *in vitro*-cultured neurons (Nouspikel et al., 2006). Interestingly, other evidence suggested the function of GG-NER in lesion removal in postmitotic cells (de Boer et al., 2002; Lans and Vermeulen, 2015). It is still conceivable that GG-NER reduces the chance of Pol II stalling at lesions on the non-transcribed strand.

To sum up, the tissue-specific balance between TC-NER and GG-NER may explain why apparently converging NER pathway deficiencies can cause such a dramatic phenotypic difference: in proliferating cells, GG-NER is responsible to remove the bulk of lesions throughout the genome and induces DNA damage signaling to dampen replication of damaged DNA, whereas in postmitotic cells, neurons in particular, TC-NER dominates the lesion removal, and all the repair mechanisms involve the elimination of the persist Pol II stalling, which is lethal to cells.

Thereby, it is quite possible that the mutation of the proteins working in TC-NER leads to neurological abnormalities. As we all have known, CS is caused by dysfunction of CSB and CSA, which are the key factors of the initiation of TC-NER. In addition, the proteins shared by both TC-NER and GG-NER are also probably related to neurological abnormalities if they mutated. In line with that, mutations in ERCC1 (XPF), ERCC5 (XPG), and the components of TFIIH, including XPD, XPB, and TF2H5, lead to cerebro-oculo-facio-skeletal syndrome 4 (COFS4) (Kashiyama et al., 2013), xeroderma pigmentosum complementation group G (XP-G), COFS3 (Zafeiriou et al., 2001; Emmert et al., 2002; Drury et al., 2014), XP-D (Broughton et al., 1995), XP-B (Oh et al., 2006), and trichothiodystrophy 3 (TTD3) (Giglia-Mari et al., 2004), respectively, and all these disorders are reported to share a same feature—CS, in some cases. The fact that mutations of converged proteins in TC-NER and GG-NER caused CS in some cases rather than all, and severely obstructed development in most cases, also indirectly proved that GG-NER is the key player in proliferating cells and the backup in neurons. In turn, CS presented developmental impair features but skin cancer, indicating that TC-NER could also be activated in mitotic cells. But one problem arises that UVSSA, cofactor of RNA Pol II in TC-NER, was identified as a causal gene of several cases of UV-sensitive syndrome (UV^S^), which is lacking the neurological pathology of CS but their cells are UV-sensitive and defective in TC-NER (Nakazawa et al., 2012; Schwertman et al., 2012; Zhang et al., 2012). In that case, whether CS is a repair syndrome remains a question.

### Transcription-Coupled Homologous Recombination—Another Scenario for CS?

As we have depicted that most of the oxidative modification on bases could be bypassed by RNA Pol II and repaired by BER in neuron cells. Nevertheless, in some cases, the surge of oxidative DNA lesions, which loads too much stress to the BER system, frequently results in the DSBs. DSB lesions could be efficiently yet highly mutagenic restored by NHEJ in neurons, as described above. However, the actively transcribed genes adopt an alternative—TC-HR, to efficiently and accurately rejoin DSBs. The repair rate is 8-folds more efficient when the nicks are located on the transcribed strand as opposed to nicks on the non-transcribed strand (Davis and Maizels, 2014) (Figure 4C).

During TC-HR, RNA Pol II stalls at the lesion, the DNA–RNA hybrid structure recruits CSB. Then, the interaction between RNA Pol II and CSB initiates the TC-HR and provides a scaffold for the HR factors, such as Rad 52 and Rad 51C, which directly interact with CSB. Thus, CSB, probably together with RNA Pol II, seems to possess distinct function in discrimination of DNA lesions and initiation of different repair pathways when encountering different types of lesions, such as CPDs or DSBs. The differential interplay with NER and HR factors requires precise regulation to achieve lesion-specific engagement of repair factors. Understanding this regulatory switch may provide critical insights into the neuronal defect observed in CS and other HR-deficient patients.

In any case, TC-HR repair may be a critical mechanism of DSB repair that protects the sequence integrity of Pol II-transcribed genes, especially in cells during G0/G1 phases, especially for neurons with long-term viability. But it is also worth noting that in the absence of precise processing and regulation mechanism, the “template” role of RNA remains unclear and should be pursued in future studies.

### The Fate of RNA Pol II Blocked by the DNA Lesions

In DNA repair, RNA pol acts as sensor and scaffold, so it only works in the early stage of transcription-related DNA repair. Next, what is the fate of RNA Pol II? The fate of the Pol II paused at a lesion is largely determined by the feature of the lesion—it bypasses the structurally subtle lesions then resumes the elongation, probably with the help of Cockayne syndrome B protein (CSB), which is proposed to promote forward movement of Pol II (Xu J. et al., 2017). While in the case of bulky and structurally distorting DNA lesions on the template strands, such as CPDs, which are insurmountable by CSB, more complicated models direct the removal of RNA Pol II: CSB removes Pol II from the lesions (Cheung and Cramer, 2011); transcription elongation factor S-II (TFIIS) backtracks the Pol II (Kettenberger et al., 2004; Zatreanu et al., 2019); Additionally, RPB1, the largest subunit of Pol II, is polyubiquitinated and degraded by proteosome (Wilson et al., 2013; Nakazawa et al., 2020). All the release of Pol II has been proposed to expose and report the lesion to DNA damage patrol system (Citterio et al., 2000), and to make the DNA lesion accessible to the subsequent TC-NER components.

It should be noticed that the ubiquitylation and degradation of Pol II is a pivotal event to regulate global shutdown of transcription and the initiation of DNA repair. As a matter of fact, Pol II, CSB, and UVSSA undergo ubiquitylation state changes in response to DNA damages. RPB1 is predominantly ubiquitinated at single lysine site, K1268 by cullin-RING ligase (CRL) and then followed by the mono-ubiquitination of UVSSA at K414. In contrast to Pol II and UVSSA, CSB is deubiquitinated by USP7 to reverse its ubiquitination state in TC-NER.

The physiological significancxe of Pol II degradation may rely on two aspects: for one aspect, the ubiquitination and degradation of RPB1 activate TC-NER and, in parallel, prevents prolonged transcription arrest after sending the TC-NER initiation signal (Nakazawa et al., 2020). In this model, upon RNAPII stalling at a lesion, CSB/CSA/CRL complex binds to Pol II and contribute to the ubiquitylation of RPB1, which is essential for its interaction with UVSSA. The sequential ubiquitination of Pol II and UVSSA coordinates the recruitment of TFIIH (Nakazawa et al., 2020). TFIIH is supposed to displace UVSSA and Pol II to provide access for core NER factors to incise the damaged strand (Schärer, 2013). This model highlighted the importance of ubiquitination of Pol II in TCR initiation and is proved by the evidence that cells in with RPB1-K1268R, one mutant that cannot be ubiquitinated, exhibited several defects in TC-NER triggered by a stalled RNAPII, including the attenuated transcription shutdown program, suppressed assembly of TC-NER complexes, defective recovery of RNA synthesis (a hallmark of TC-NER completion), and hypersensitivity to UV irradiation of the cells (Bernecky et al., 2016; Nakazawa et al., 2020; Tufegdzic Vidakovic et al., 2020). In line with the results from cell lines, RPB1^K1268R/K1268R^/XpA^−/−^ double-mutant mice display progressive neurodegenerative phenotype, underscoring the importance of RNA Pol II removal (in contrast to humans, the full Cockayne syndrome phenotype only manifests in mice in the absence of GG-NER), suggesting that the absence of RNAPII degradation, caused by the absence of either RPB1 ubiquitylation or CSB, is the leading cause for Cockayne syndrome, and explaining the CS-like aging-related phenotypes by a deficiency in RNA Pol II processing and prolonged transcription arrests under a high load of endogenous DNA damage rather than a compromised DNA repair activity associated with TC-NER (Nakazawa et al., 2020).

Another meaning of Pol ubiquitination is probably that the ubiquitination and degradation alleviate the accumulation of RNA Pol II at the lesions and release other subunits except RPB1 to the RNA Pol subunits pool(Tufegdzic Vidakovic et al., 2020). That is to say, insufficient RNAPII is also available for transcription, due to excessive degradation and/or stalling at lesions after damage. Correspondingly, another study has proved that RPB1-K1268R introduced into CSB cells still restored transcription restart, alleviating at least some of the transcription defects in CSB cells and transcribing a series of short genes and corresponding proteins (Tufegdzic Vidakovic et al., 2020). This theory could more rationally expound the pathology underlying CS being caused by a transcription defect and why TC-NER activity is still retained even if Pol II cannot be ubiquitinated (Lommel et al., 2000; Woudstra et al., 2002).

In summary, though RNA pol responds differently to various DNA lesions, yet it mainly serves as a sensor and initiator of DNA damage repair, there have been no reports of RNA pols participating in TC-NER in human cells.

### The Clinical Consequences of RNA Pol II Mutation

Despite the important role of RNA Pol II in maintaining the normal physiological process of cells, the direct mutation of RNA Pol II subunits has been rarely implicated in human disease thus far.

In a study using large-scale sequencing, sixteen individuals harboring *de novo* heterozygous variants in POLR2A, encoding RPB1, the largest subunit of Pol II, showed neurodevelopmental syndrome characterized by profound infantile-onset hypotonia and developmental delay, namely, neurodevelopmental disorder with hypotonia and variable intellectual and behavioral abnormalities (NEDHIB) (Haijes et al., 2019). As the CTD of RPB1 mediates the interaction between RNA Pol II and various general and specific transcription factors and regulators, we cannot attribute the role of Pol II in DNA repair to this neuronal defect.

The mutations of CSB and CSA, which fulfill key role in TC-NER as a coupling factor that attracts downstream NER proteins, are reported to be associated with most of Cockayne syndrome (CS) cases, characterized by progressive growth failure, microcephaly, mental retardation retinal degeneration, sensorineural deafness, and photosensitivity (Hafsi and Badri, 2022). The reason that CS shows some overlaps with certain forms of xeroderma pigmentosum, a very rare skin disorder which is primarily caused by the mutation of Xeroderma pigmentosum complementation group A (XPA) and shows the most severe skin symptoms and progressive neurological disorders in some cases (Lehky et al., 2021) may be because XPA works both in GG-NER and TC-NER.

### Is There a Connection Between RNA Pol III Mutation-Related Neurodegenerative Disorder and Homologous Recombination?

RNA Pol III is responsible for the synthesis of 5S rRNA, tRNAs, and various other small non-coding RNAs. Though thousands of small non-coding RNA in the transcriptome profile of RNA Pol III, for example, the Alu RNA, are reported to be involved in neurodegenerative diseases (Renoux and Todd, 2012; Sosińska et al., 2015; Polesskaya et al., 2018; Fagan et al., 2021), less evidence describes a direct role of RNA Pol III in the pathogenesis of diseases (Lata et al., 2021). There raises the question that why the mutation of RNA Pol III subunits leads to a spectrum of neuro-dysfunction. As the largest and most complex RNA polymerase in eukaryotes, RNA Pol III is composed of 17 subunits. Whereas, the mutations in the subunits POLR3A, POLR3C, POLR3E, and POLR3F are associated with susceptibility to varicella zoster virus-induced encephalitis and pneumonitis, the distinct mutations in the POLR3A, POLR3B, POLR1C, and POLR3K subunits cause a spectrum of neurodegenerative diseases, which includes the most notably hypomyelination leukodystrophy (Lata et al., 2021). Some pathophysiological hypotheses propose that the Pol III subunit mutations impair the biogenesis of the Pol III complex and the downstream transcriptome, for example, decrease the tRNA transcripts, which further leads to a global alternation of RNA profile. In line with the hypothesis, some recent researches revealed that mutations in genes important for protein translation, such as those encoding for tRNA-aminoacyl synthetases, including DARS1 (Taft et al., 2013), EPRS1 (Wolf et al., 2014), and RARS1 (Mendes et al., 2018, 2020), resulted in hypomyelination symptom. This raises the possibility that reduced availability of the corresponding aminoacyl-tRNA through Pol III or tRNA-synthetase mutations is particularly detrimental to the CNS, which have a lower threshold than other tissues for tolerating hypofunction of these enzymes. However, these hypotheses are explanation from the aspect of physiological function rather than the molecular mechanism illustration, and the precise mechanism remains to be clarified.

Recently, the role of RNA Pol III in HR shed a new light to us in understanding the relationship between RNA Pol III mutation and the neurodegenerative disorders. In HR, RNA Pol III catalyzes the synthesis of RNA strand in the RNA-DNA, which is a repair intermediate to protect the 3'-ssDNA overhangs of the DSBs (Ohle et al., 2016). It is plausible that RNA Pol III does the same in TC-HR, so far as to process the template RNA strand to so as to maximize the fidelity of DSB repair at critical regions of the genome in neurons with high fidelity, compared to NHEJ (Welty et al., 2018). In that case, there may exist the collaboration, or perhaps competition of RNA Pol II and Pol III in the nicks of actively transcribed strand, before the initiation of TC-HR in the neuron cells.

## Mutual Interaction Between Polymerases Mediated Repair Pathways and Trinucleotide Repeat (TNR) Expansion—A Vicious Loop on the Pathogenesis and Outcome of TNR Expansion Diseases

Trinucleotide repeat expansions are associated with more than 40 neurodegenerative diseases. Although the mutational mechanisms are similar, the repeated DNA sequences occur in different genomic contexts, in dividing and non-dividing cells, and are tissue-, cell-, and disease-specific (Orr and Zoghbi, 2007). However, most of these TNR expansion diseases share a common feature, ataxia with prominent cerebellar degeneration in clinical, whereas DNA damage response and DNA repair aberrance in genetic, especially the Huntington's disease (HD) and multiple spinocerebellar ataxias (SCAs) (Martins et al., 2014; Lee et al., 2015; Bettencourt et al., 2016). To intuitively understand the relationship between TNR disease and DNA repair deficiency, here, we listed some well-known TNR expansion diseases and their involved DNA repair pathways (Table 2).

**Table 2:** Characteristics of selected disease-causing repeat loci.

**Neurological abnormalities**	**Disease**	**Gene**	**Repeat sequence**	**Somatic expansion**	**DNA repair factors involved in diseases**	**Replication and repair pathways**	**Neurological abnormalities caused by impaired pathways**
Autism; Intellectual Disability (ID) Syndrome (Hagerman et al., 2017)	Fragile X syndrome/ ataxia syndrome	*FMR1*	CGG	M, H	N	mRNA translation	N
Action tremor; Bradykinesia; Cerebellar signs, including ataxia; Hyperreflexia; Paucity of movement; Babinski present; Decreased tone; Psychiatric symptoms; Focal dystonia; Dementia; Incontinence (Klockgether et al., 2019)	Spinocerebellar ataxia 12	*PPP2R2B*	CAG	Rare	PP2A	• NHEJ (Wang et al., 2009) • HR (Ambjørn et al., 2021)	A-T (Li et al., 2012; Teive et al., 2015): Movement disorders and motor disturbances; Cerebellar ataxia; Oculo-cutaneous telangiectasia
Focal atrophy; Motor neuron degeneration; Muscle weakness; Paralysis (Abramzon et al., 2020)	Frontotemporal dementia and amyotrophic lateral sclerosis	*C9ORF72*	GGGGCC	H	• TP53 (Maor-Nof et al., 2021) • P62, RNA Pol (Haeusler et al., 2014) • ATM (Walker et al., 2017)	oxidative stress DNA repair (Lopez-Gonzalez et al., 2016; Yuva-Aydemir et al., 2018)	HD (Hyeon et al., 2021): Motor impairment; Cognitive impairment; Depression; Anxiety AD (Shen et al., 2021): Dementia
Ataxia; Choreoathetosis; Dementia (Sugiyama et al., 2020)	Dentatorubral-pallidoluysian atrophy	*ATN1*	CAG	H, M	• P21 • P62	NHEJ (Deguise et al., 2016)	N
Involuntary movement; Cognitive impairment; Depression; Anxiety, Neuropsychiatric changes, Physical pain (Underwood et al., 2017)	Huntington's disease	*HTT*	CAG	H, M	• FAN1, MLH3, MSH3, *DHFR* (Ciosi et al., 2019) MLH1 (Lee et al., 2015) • APEX1 (Mollica et al., 2016) • PNKP (Gao et al., 2019)	• Oxidative DNA damage repair (Ayala-Peña, 2013) • BER • MMR (Goold et al., 2021) • TC-NER • NHEJ	Cerebellar ataxia; Axonal neuropathy; Cognitive impairment; Hypercholesterolemia; Hypoalbuminemia; Hyperkinetic dyskinesia; Microcephaly.
Muscular dystrophy; dysarthria; dysphagia; muscle atrophy (Hashizume and Katsuno, 2018)	Spinal and bulbar muscular atrophy	*AR*	CAG	H, M	• IGF (Hashizume and Katsuno, 2018) • HDAC6 (Grunseich et al., 2014) • P53 (Malik et al., 2019)	• Mitochondrial dysfunction • Transcription dysfunction (Malik et al., 2019) • BER (Vasquez et al., 2020)	Parkinsonism; Flexed posture; Extremity hyperreflexia; Dementia; Polyproteinopathy
Progressive ataxia; Cerebellar atrophy; axonal sensor motor neuropathy; Hypercholesterolemia; cognitive impairment (Martins et al., 2017)	Spinocerebellar ataxia 1	*ATXN1*	CAG	H, M	• ATM (Suart et al., 2021), MRE11A, MSH2, WRN (Spence and Wallihan, 2012) • RpA1 (Bosso Taniguchi et al., 2016)	• ATM mediated DNA repair (Suart et al., 2021) • Mitochondrial DNA damage repair (Ito et al., 2015)	Radiosensitivity, Immunodeficiency Learning difficulties; Movement disorders and motor disturbances; Cerebellar ataxia; Oculo-cutaneous telangiectasia
Symptoms of amyotrophic lateral sclerosis; Extraocular muscle palsy; Severe tremor and myoclonus (Egorova and Bezprozvanny, 2019)	Spinocerebellar ataxia 2	*ATXN2*	CAG	H, M	MTHFR (Almaguer-Mederos et al., 2020) mTORC1 (Auburger et al., 2017) TDP-43	TC-NER	Cerebellum cerebrum, neurons loss; Parkinsonism; Flexed posture; Extremity hyperreflexia; Dementia; Polyproteinopathy
Cerebellar ataxia; Extraocular muscle palsy; Dysarthria; dysphagia; Dystonia; Distal muscular dystrophy (Zhang et al., 2021)	Spinocerebellar ataxia 3	*ATXN3*	CAG	H, M	• TP53 (Liu et al., 2016) • CHK1 (Tu et al., 2017) • PNKP (Chatterjee et al., 2015; Gao et al., 2015; Chakraborty et al., 2020) • MDC1	• NHEJ (Chakraborty et al., 2020) • DSBR • NER	Cocaine syndrome, sulfur dystrophy, brain-eye-skeletal syndrome
Dysphagia; delayed progressive cerebellar ataxia; nystagmus; language disorder (Tamura et al., 2017)	Spinocerebellar ataxia 6	*CACNA1A*	CAG	Rare meiotic	ATM, APTX (Kashimada et al., 2019)	N	Movement disorders and motor disturbances; Cerebellar ataxia; Oculo-cutaneous telangiectasia
Permanent blindness, dysarthria, difficulty swallowing; brain-eye-skeletal syndrome (Klockgether et al., 2019)	Spinocerebellar ataxia 7	*ATXN7*	CAG	H, M	• FUS (Niss et al., 2021) • H2B (Ramachandran et al., 2016)	N	N
Progressive gait and limb ataxia; Auditory and optic neuropathy; Cardiomyopathy; scoliosis; Dysarthria; Dysphagia (Keage et al., 2017)	Friedreich's ataxia	*FXN*	GAA	H, M	MSH2, MSH3 (Halabi et al., 2012)	• NER (Moreno-Lorite et al., 2021) • BER (Moreno-Lorite et al., 2021) • SSBR (Moreno-Lorite et al., 2021) • MMR (Neil et al., 2021) • DSBR (Neil et al., 2021)	Peripheral axonal neuropathy; Oculomotor apraxia; Hypoalbuminemia
Sleep apnea; Central and obstructive sleep apnea (Harley et al., 1992)	Myotonic dystrophy 1	*DMPK*	CTG	H, M	N	N	N
Gait ataxia; Parkinson; language disorder (Klockgether et al., 2019)	Spinocerebellar ataxia 8	*ATXN8*	(CTA)_n_ (CTG)_n_	H	N	N	N

*Only parts of disease-causing repeat loci are included, so many rare diseases caused by expansion of repeat codons, including those giving rise to shorter polyalanine tracts are not shown. H, seen in human tissues; M, seen in mouse model tissues; N, no literature has reported relevant cases*.

*Red font: The symptom shared by TNR diseases and general symptom caused by impaired DNA pathway*.

**Figure 7:**
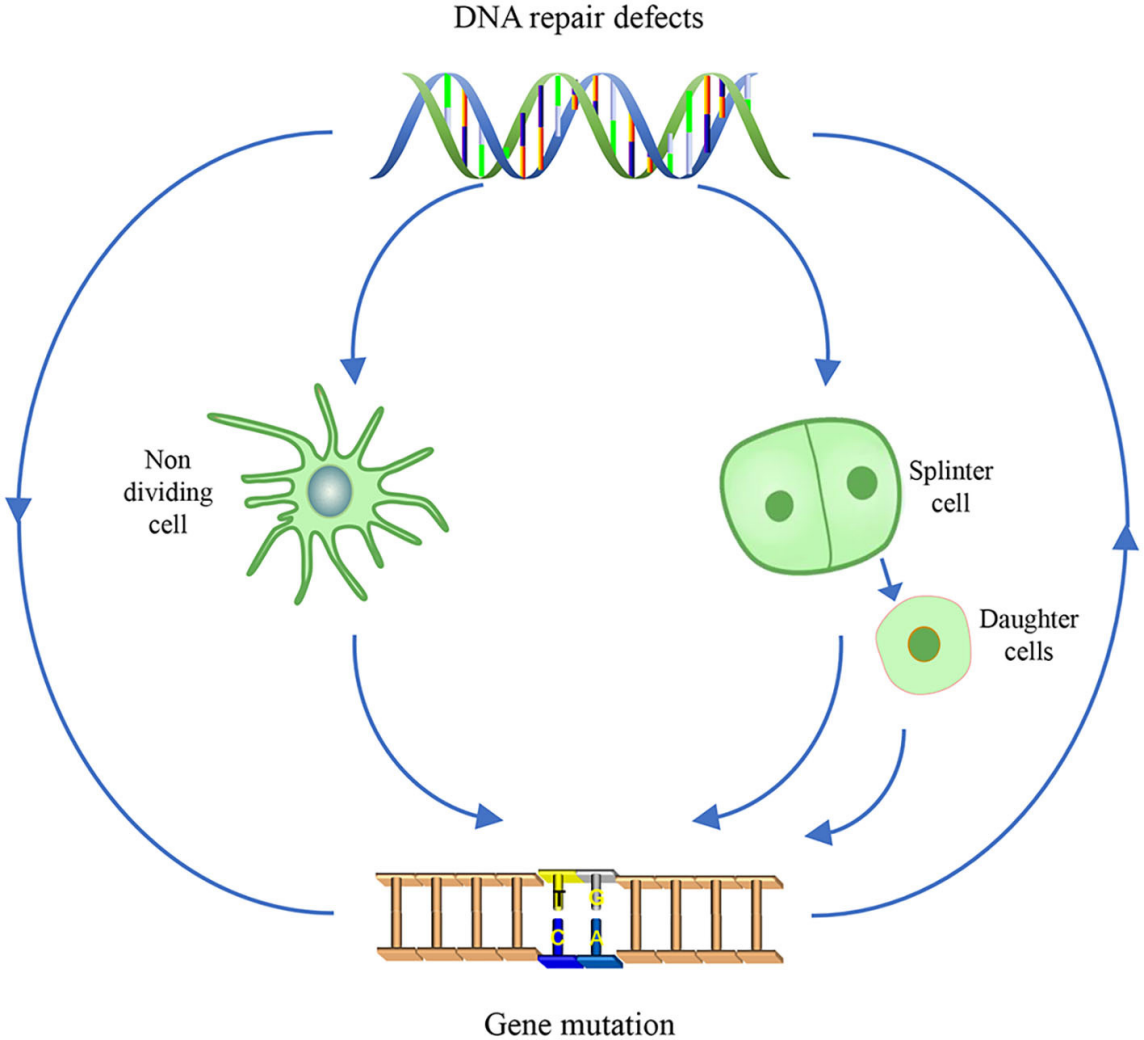
The vicious cycle of gene mutation and impaired DNA repair pathway in TNR diseases. For non-dividing cells, gene mutation disrupting the DNA repair pathway is displayed as cell senescence and apoptosis, that is the outcome of the TNR diseases. For dividing cells, the aberrant DNA repair pathway in turn exacerbates the TNR expansion. In somatic cells, the gene mutation will be passed on and accumulate as the cells proliferate; In germ cells, the aggravated gene mutation is inherited by the offspring, which will exhibit more severe TNA disease symptom in neurons system and aggravated mutagenicity in somatic cells.

It is easy to understand that the mutation of the diseases protein interrupts pathways, which exerts crucial influence on the recovery of DNA lesions in neurons, thus resulting in the neuron loss and degeneration outcomes. But still, there is a vicious loop that the disturbed DNA repair pathways in turn accelerate the mutation in proliferating cells, for example, in the stem cells, and lead to more severe TNR expansion in the next generation (Figure 7).

It is well-established that interruptions in DNA repair are important for modifying the age of onset; the most studied is HD. HD is an autosomal dominant neurodegenerative disorder that affects 1 in 7,300 people in the western population (Lans et al., 2019). Amplification of CAG repeats within exon 1 of the huntingtin (*Htt*) gene results in the expansion of polyglutamine (polyQ) residues in the N-terminus of the huntingtin protein (HTT) (Labbadia and Morimoto, 2013; Moily et al., 2017). The presence of HTT is toxic to striatal GABAergic neurons, resulting in the degeneration of the striatum.

Mutant HTT has been shown to impair NHEJ by disrupting Ku70-Ku80 heterodimer formation and Ku70-DNA interaction. Consequently, the DNA-PK complex activity was compromised. In turn, overexpression of Ku70 was shown to ameliorate the HD phenotype in R6/2 mouse models (Enokido et al., 2010; Ferlazzo et al., 2014). The impaired NHEJ manifested the neurological aberrant in CNS neurons and the TNR instability in either somatic or germ cells during replication. It is interesting that HTT impairs NHEJ probably by the mutation of protein, whereas NHEJ exacerbates the HTT mutation by direct excision on DNA.

Another example is spinocerebellar ataxias III (SCA3). SCA3 is caused by CAG trinucleotide repeat expansions that are translated into aberrant long polyQ tracts in Ataxin-3 protein (McLoughlin et al., 2020). Postmortem SCA3 disease brains exhibit significant neuronal dysfunction and neuronal cell loss spanning the CNS system. Several recessive ataxias exhibiting CNS-limited degeneration are caused by mutations in BER and SSBR repair genes, suggesting that the CNS may be exceptionally vulnerable to the disturbance to these repair pathways (Massey and Jones, 2018).

The physical function of Ataxin-3 involves in the fine-tuning of ATM and ATR-activated downstream DNA damage responses by controlling the turnover of MDC1, ChK1, and p53 (Liu et al., 2016; Pfeiffer et al., 2017; Tu et al., 2017). Importantly, Ataxin-3 also interacts with PNKP, a key enzyme in BER and NHEJ (Chatterjee et al., 2015; Gao et al., 2015). A more recent study revealed that Ataxin-3 and PNKP also stably associate with TC-NER complexes composed of RNA Pol II, CBP, and surprisingly HTT protein (Gao et al., 2019), indicating a role of Ataxin-3 in TC-NER. The mutation of Ataxin-3 disrupts the normal processes of DNA repair, leading to neuron loss in CNS, as well as the abnormal activation of non-neuronal cells in brain (Rüb et al., 2002; Yang et al., 2017). It is possible that the disrupted DNA repair pathway contributes to the pathogenesis of SCA3.

Notably, in turn, the aberrant DNA repair pathways retaliate in the germ cells, which will generate next generation with longer CAG repeat expansion. In brief, the TNR expands during replication and repair of DNA lesions produced by oxidative stress. Several DNA repair mechanisms, including mismatch repair (MMR), TC-NER, and BER, have been proposed to be involved in TNR somatic and germline expansion (Kovtun and McMurray, 2001; Salinas-Rios et al., 2011). Unfortunately, SCA3 involves all these DNA repair pathways. BER may be directly participated in TNR expansion, because silencing of Ogg1 gene abolished age-dependent neuronal CAG repeat expansion in a HD mouse model (Kovtun et al., 2007). BER-mediated TNR expansion involves DNA lesion-containing strand slippage, hairpin formation in the CAG repeats region, and inhibition of pol β-FEN1 coordination (Liu et al., 2009). Generally, the coordination of pol β and FEN1 could be guaranteed by PCNA, HMGB1, and PARP-1 through protein–protein interactions in LP-BER. However, once the coordination fails, CAG repeats could escape from LP-BER excision, and spontaneous hairpin formation may occur, coupled with multinucleotide gaps. DNA synthesis to fill the multinucleotide gaps and ligation of the hairpin structures would lead to TNR expansion. Under this context, a vicious cycle starting from the mutation of Ataxin-3, experiencing DNA repair defect, and ending with aggravated mutation induced by DNA repair defect is formed.

## Concluding Remarks and Perspective

The bedrock for the maintenance of genomic integrity lies in the exquisite fidelity with which the genome is replicated by DNA polymerases, as well as in the mechanisms for DNA repair. Thus, the study of the human DNA polymerases is highly pertinent in the context of the constellation of factors that maintain genomic stability.

We have provided a general overview on the polymerases, which mediate the DNA repair events that are prone to occur in non-dividing cells. The RNA polymerases are more likely to work during the initiation stage of repair. They serve as DNA damage sensors, and scaffolds in docking other repair factors. To date, no evidence has announced the activity in DNA lesions removal of the RNA polymerases. DNA polymerases are involved in the late stage of repair. After repair initiated by other proteins, they work to process the break ends and fill in the gap. The synthesis and fidelity feature of these polymerases may determine what they do in DNA repair, but when and how they carry out repair task is determined by their coordination proteins. The incoordination between polymerase and their partners during DNA repair probably contributes to the source of the vicious cycle of TNR diseases.

To date, the main frame of DNA repair pathways is well-established. As more and more novel functions of the proteins and enzymes are discovered, new accessory factors engage in the DNA repair repertoire. These new proteins fine-tune the canonical machinery, depending on the cell type and cell cycle (Ghosh and Raghavan, 2021). This will promote our understanding on how the cells determine which pathway to take and when it should be turned on. On this basis, the biological relevance between DNA repair pathways and the pathogenesis of neurodegenerative diseases will constantly advance.

But still, there are also remarkable breakthroughs on DNA repair mechanism. Previously, NHEJ is considered as the only way to restore DSBs in non-dividing cells. It is very efficient in reattachment of broken DNA ends, but unluckily with low fidelity. As neurons suffer from ROS insult with high frequency all the time, it is irrational that neurons only keep such a mutagenesis way to maintain their genome stability, till RNA-driven DNA repair pathway was discovered (Meers et al., 2016, 2020). The transmission of genetic information from RNA to DNA, though reverse the Central Dogma in mammalian cells, complements the shortcoming of both NHEJ and HR and provides abundant homologous substrate that is present in the form of transcript RNA and may have a substantial role in genome stability. To view this mechanism more optimistically, it is possible that one targeted gene therapy strategy may be developed using RNA as a template to permanently correct the gene mutations in non-dividing cells, shedding new light to inherited neuronal diseases.

## Author Contributions

All authors listed have made a substantial, direct, and intellectual contribution to the work and approved it for publication.

## Funding

This work was funded by the National Key Research and Development Program of China (2018YFA0108500), National Natural Science Foundation of China (31801198, 81871029, 81921006, 32070780, and 82030033), Natural Science Foundation of Hebei Province (C2021203004), and Natural Science Foundation of Beijing (5181001).

## Conflict of Interest

The authors declare that the research was conducted in the absence of any commercial or financial relationships that could be construed as a potential conflict of interest.

## Publisher's Note

All claims expressed in this article are solely those of the authors and do not necessarily represent those of their affiliated organizations, or those of the publisher, the editors and the reviewers. Any product that may be evaluated in this article, or claim that may be made by its manufacturer, is not guaranteed or endorsed by the publisher.
